# First Evidence for the Disease-Stage, Cell-Type, and Virus Specificity of microRNAs during Human Immunodeficiency Virus Type-1 Infection

**DOI:** 10.3390/medsci4020010

**Published:** 2016-05-13

**Authors:** Lauren Fowler, Viviane Conceicao, Suneth S. Perera, Priyanka Gupta, Choo Beng Chew, Wayne B. Dyer, Nitin K. Saksena

**Affiliations:** 1 Westmead Millennium Institute & Westmead Hospital, University of Sydney, Westmead, Sydney, NSW 2145, Australia; lauren_fowler@live.com.au (L.F.); vickanc@hotmail.com (V.C.); perera.suneth@gmail.com (S.S.P.); pgup7905@uni.sydney.edu.au (P.G.); 2 Research and Development, Australian Red Cross Blood Service, 17 O’Riordan Street, Alexandria, Sydney, NSW 2015, Australia; choobengchew@hotmail.com; 3 ICPMR, Department of Virology, Westmead Hospital, Westmead, Sydney, NSW 2145, Australia; Wayne.dyer@sydney.edu.au

**Keywords:** CD4+ T-cells, CD8+ T-cells, micro-RNA, human immunodeficiency virus, acquired immunodeficiency syndrome, non-progression, gene expression

## Abstract

The potential involvement of host microRNAs (miRNAs) in HIV infection is well documented, and evidence suggests that HIV modulates and also dysregulates host miRNAs involved in maintaining the host innate immune system. Moreover, the dysregulation of host miRNAs by HIV also effectively interferes directly with the host gene expression. In this study, we have simultaneously evaluated the expression of host miRNAs in both CD4+ and CD8+ T-cells derived from HIV-positive (HIV+) individuals (viremic and aviremic individuals while receiving highly active antiretroviral therapy (HAART), therapy-naïve long-term non-progressors (LTNP), and HIV-negative (HIV–) healthy controls. miRNAs were run on Affymetrix V2 chips, and the differential expression between HIV+ and HIV− samples, along with intergroup comparisons, was derived using PARTEK software, using an FDR of 5% and an adjusted *p*-value < 0.05. The miR-199a-5p was found to be HIV-specific and expressed in all HIV+ groups as opposed to HIV– controls. Moreover, these are the first studies to reveal clearly the highly discriminatory miRNAs at the level of the disease state, cell type, and HIV-specific miRNAs.

## 1. Introduction

MicroRNAs (miRNAs) are small (21–22 nt), non-coding RNA fragments found in many organisms, from plants to humans, which function to negatively regulate gene expression. In humans, roles for miRNA have been identified in metabolism, growth, and development. Dicer, a protein critical for miRNA processing, has been associated with proper development of the limbs, lungs, and hair follicles and in T-cell differentiation [1,2]. Dysregulation of human miRNA has been implicated in the pathogenesis of many cancers [3]. As negative regulators of gene expression, miRNAs can function as tumor suppressors that, if dysregulated, can augment the chance of cancer development [4]. For example, a chromosomal deletion associated with the development of chronic lymphocytic leukaemia (CLL) causes a loss of intrinsic miRNA tumor suppressors [5]. Currently, over 800 miRNAs have been identified in the human genome with diverse functions. miRNAs function by binding to partially complementary sites in the messenger RNA (mRNA) of other genes, inhibiting the production of the proteins synthesized from these genes. A single miRNA can interact with hundreds and thousands of genes in regulating various physiological processes, thus adding to the complex regulation of the human genome.

Viruses are known to subvert and manipulate the human gene machinery, and miRNAs are no exception. Host miRNAs are dysregulated by viruses, and both DNA and RNA viruses have been found to encode and express miRNAs in the infected host, demonstrating the importance of the role of host- and virus-encoded miRNAs in host-virus interactions. The miRNA expression profile of cells is altered in both clinical PBMC samples and cultured cells. Cells transfected with an infectious HIV-1 strain have significantly altered miRNA profiles compared to control cells [6]. In 2008, Houzet *et al.* showed that the vast majority of host miRNAs are downregulated in HIV infection of peripheral blood mononuclear cells (PBMCs). When individuals were separated into groups based on their T-cell counts and viral loads (VLs), specific miRNA “profiles” could be seen for each of the classes. Furthermore, many miRNA changes in patient cells could not be accounted for by infection alone, indicating a complex role for miRNA in gene regulation [7].

In 2007, Huang *et al.* showed overexpression of host miRNAs in resting T-cells that target sequences in the 3′ end of HIV-1 RNA, silencing viral mRNA and enforcing latency [8]. Furthermore, Witwer *et al.* showed that PBMC miRNA profiles could distinguish elite suppressors (ES) and uninfected controls from viremic HIV-1 infected patients [9]. Their results demonstrated correlations between miRNA expression, CD4+ T-cell count and viral load. Some miRNAs found to differ in expression have previously been shown to correlate with HIV-1 latency, including miR-29s, miR-125b, and miR-150. Their analysis identified several miRNAs that have not been previously described in association with HIV infection, including miR-31, which distinguishes controls and ES and regulates a protein with implications for T-cell differentiation. Although this study has also shown that HIV-1-positive ES are characterized by a PBMC miRNA profile that in general resembles that of uninfected individuals, they also reiterate that the ES, on the basis of miRNA expression, are a heterogeneous group. This suggests that different mechanisms, shaped or marked by different miRNA expression patterns, underlie sustained and durable control in therapy-naïve HIV-infected individuals. In a recent International AIDS Society (IAS) meeting, Zhu *et al.* showed a set of 18 differentially expressed miRNAs, which could identify the outcome of HIV disease at the chronic stage more accurately. Six out of 18 miRNAs were significantly related to faster rate of CD4+ T-cell decline [10]. Studies of larger cohorts of individuals are needed to address miRNA specific to different stages of HIV disease and explain the underlying genomic basis of natural control of HIV in therapy-naive ES.

Since all the studies to date have been performed on whole PBMC or tissue, we endeavored to address disease- and cell-type-specific miRNAs and their role in HIV pathogenesis. We have adopted a novel approach for this study, which simultaneously analyzes miRNAs from the CD4+ and CD8+ T-cells from viremic, aviremic BDL patients, and elite controllers. This study is unique in showing the HIV disease-stage and cell-type specificity of miRNA during HIV infection and its natural control in elite controllers.

## 2. Results

### 2.1. Patient Samples Used in Microarray Analysis

Patients were classed into disease groups based on their HIV plasma viral load (VL) and also the antiretroviral drug treatment, as shown in Table 1. Prior to the microarray analysis, RNA quality and integrity was checked with an Agilent Bioanalyzer. All RNA samples with an RNA integrity number (RIN) above 8 were deemed appropriate for microarray analysis. The results are shown below in Table 1 for each sample and specific cell types analyzed.

### 2.2. Assessment of the Quality and Integrity of the Dataset by Principal Component Analysis (PCA)

**Figure 1:**
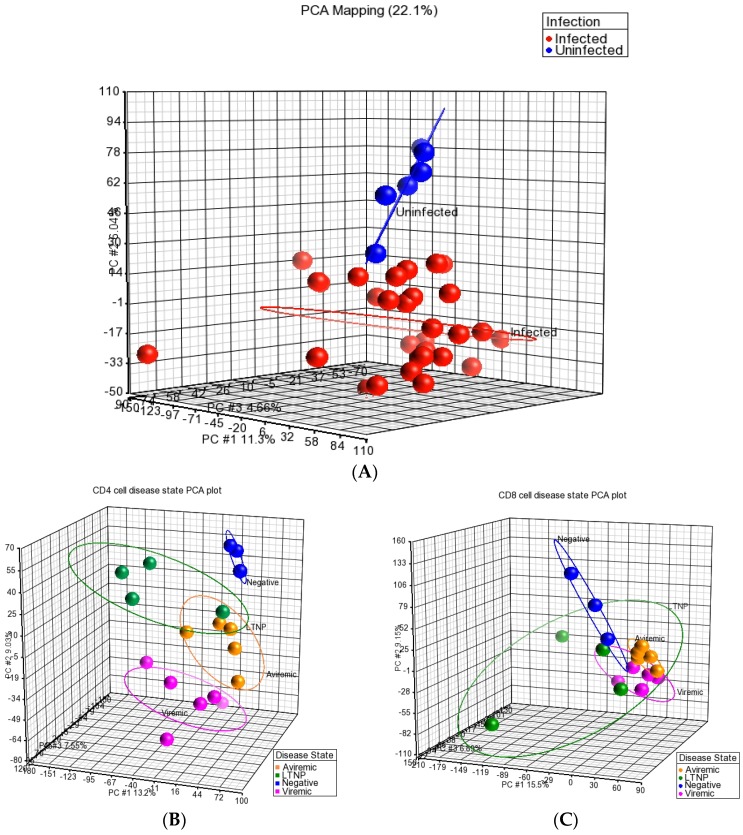
(**A**) Principal component analysis (PCA) of samples highlighting concordance and clustering of all infected cell samples, compared to uninfected samples. *X* Axis (Component 1) = 11.3%, *Y* Axis (Component 2) = 6.04%, *Z* Axis (Component 3) = 4.66%. **Red** = HIV-positive (HIV+), **Blue** = HIV-negative (HIV–), healthy donors; PCA of CD4+ (**B**) and CD8+ (**C**) T-cells respectively, highlighting disease group concordance. **Pink** = viremic, **Yellow** = aviremic, **Green** = LTNP, **Blue** = negative. Viremic *n* = 6, aviremic *n* = 5, LTNP *n* = 4, negative *n* = 3 for each cell type. (**B**) *X* axis (Component 1) = 13.2%, *Y* axis (Component 2) = 9.03%, *Z* axis (Component 3) = 7.55%. (**C**) *X* axis (Component 1) = 15.5%, *Y* axis (Component 2) = 9.15%, *Z* axis (Component 3) = 6.89%.

**Figure 2:**
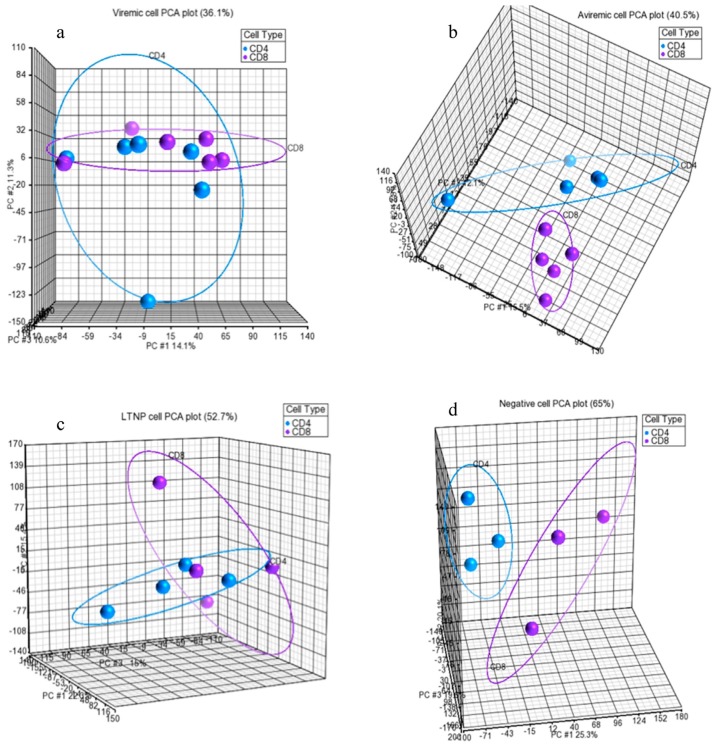
PCA for each disease state, comparing CD4+ and CD8+ T-cells. Analysis of CD4+ and CD8+ cells from patient groups viremic (**a**); aviremic (**b**); LTNP (**c**) and negative (**d**). Azure = CD4+ T-cells, **Purple** = CD8+ T-cells. (**a**) Viremic CD4+ (*n* = 6) *vs.* CD8+ (*n* = 6). *X* Axis (Component 1) = 14.1%, *Y* Axis (Component 2) = 11.3%, *Z* Axis (Component 3) = 10.6%. (**b**) Aviremic CD4+ (*n* = 5) *vs.* CD8+ (*n* = 5). *X* axis (Component 1) = 15.5%, *Y* axis (Component 2) = 12.8%, *Z* axis (Component 3) = 12.1%. (**c**) LTNP CD4+ (*n* = 4) *vs.* CD8+ (*n* = 4). *X* axis (Component 1) = 22.3%, *Y* axis (Component 2) = 15.4%, *Z* axis (Component 3) = 15%. (**d**) Negative CD4+ (*n* = 3) *vs.* CD8+ (*n* = 3). *X* axis (Component 1) = 25.3%, *Y* axis (Component 2) = 20.1%, *Z* axis (Component 3) = 19.6%.

Further, upon data retrieval from microarray analysis, the quality and integrity of the miRNA dataset were also assessed using the principal component analysis (PCA) available in the Partek Genomics Suite. Data in Figure 1A, shows the analysis of all HIV-infected groups against the HIV-negative (HIV–) healthy donors. This data reflects the high integrity of samples through a clear distinction between HIV-infected and uninfected healthy donors, deeming these samples fit for inclusion in downstream analysis. Following the broad HIV-positive (HIV+) *vs.* HIV− analysis (Figure 1), we examined the inter-group contrasts using the PCA for their integrity based on the cell types (CD4+ and CD8+ T-cells), as shown in Figure 1B,C. Again, excellent segregation was apparent for all four contrasts (long-term non-progressor (LTNP), aviremic, viremic and HIV– groups) in both CD4+ and CD8+ T-cells. From these data, it is clear that the miRNA profiles of the four disease states examined were distinct and separable. One interesting observation was that the segregation of groups based on cell phenotype was better resolved for all four groups examined in CD4+ T-cells (Figure 2a–d). In contrast, the CD8+ T-cells, although indicating segregation of all four groups, showed considerable closeness between viremic, aviremic, and LTNP groups, which was expected, as these three groups were HIV+. Taken together, the data represented in Figure 1, suggest unambiguous data integrity between contrasts, which provided a solid platform to study differentially expressed (DE) miRNA between different HIV disease groups.

The underlying reasons for this distinction between CD4+ and CD8+ T-cells in their ability to segregate HIV disease and non-disease groups remain unclear. PCA was also used to determine cell specificity in relation to disease group by directly comparing the CD4+ and CD8+ T-cells of each disease state. This analysis indicated a clear segregation of CD4+ and CD8+ T-cell type based on disease state, implying cell-specific functional segregation during HIV disease stages (Figure 2a–c) and in a healthy state, as can be seen in Figure 2d. Although good segregation of CD4+ and CD8+ T-cells was seen based on disease group for viremic (a), aviremic (b), and negative (d) comparisons, the data shown for the LTNPs in Figure 2c show a close relationship and little segregation between CD4+ and CD8+ T-cells, suggesting a possible functional significance of CD4+ and CD8+ T-cells and cooperation between them in mounting sustained response during natural control of HIV in the LTNPs.

### 2.3. Frequency Histograms

**Figure 3:**
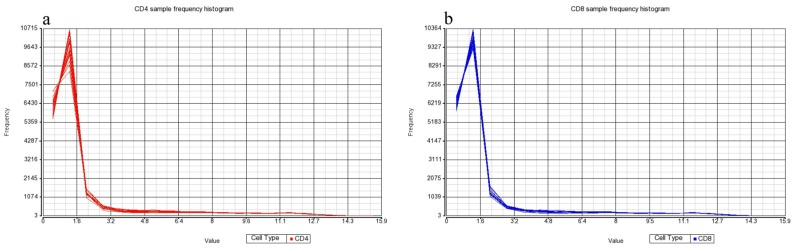
Frequency histograms of CD4+ (**a**) and CD8+ (**b**) cell samples, showing the concordance of data from each T-cell type. CD4+/CD8+ *n* = 18.

Frequency histograms representative of the CD4+ and CD8+ T-cell datasets (Figure 3a,b) show that normalization applied by the Partek Genomics Suite was effective and that there was negligible patient-to-patient variability. High concordance between all 18 samples based on CD4+ (Figure 3a) and CD8+ T-cells was evident (Figure 3b), which supports the validity of the processed and normalized data for further analysis.

### 2.4. Differential Expression of miRNAs between HIV Positive Samples and Negative Controls

Upon a quality and integrity check using various algorithms discussed above, we analyzed differential miRNA expressions between disease groups and disease groups based on cell types. Heat maps, coupled with hierarchical clustering, were used throughout to visualize the validity and integrity of differential expressions between each contrast.

**Figure 4:**
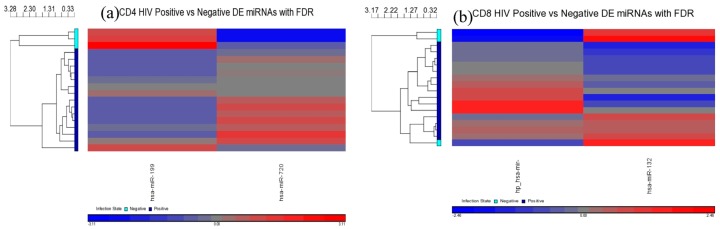
(**a**,**b**) Heat maps indicating differentially expressed (DE) microRNAs (miRNAs) in HIV+ CD4+ (**a**) and CD8+ (**b**) T-cells compared to negative controls. In each case, uninfected *n* = 3 and infected *n* = 15.

**Figure 5:**
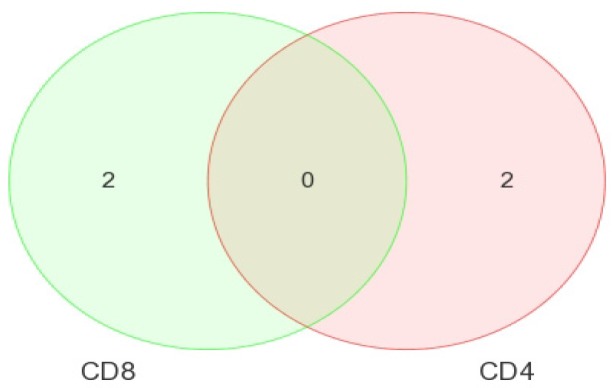
Venn diagram showing miRNAs specific to CD4+ and CD8+ T-cells with the absence of any overlapping miRNAs between these two cell types. Uninfected *n* = 3, and HIV-infected *n* = 15.

In the first contrast, all of the HIV+ groups (viremic, aviremic, LTNP) were taken together and compared against the HIV– healthy donors using the false discovery rate (FDR). Two DE miRNAs were found specific to CD4+ T-cells (miR-199a-5p and miR-720) (Figure 4a and Figure 5), and two specific to the CD8+ T-cells (miR-941-3s and miR-1323) (Figure 4b and Figure 5, Table 2), with significant fold changes (FCs) supported by strong *p*-values (Table 2).

**Figure 6:**
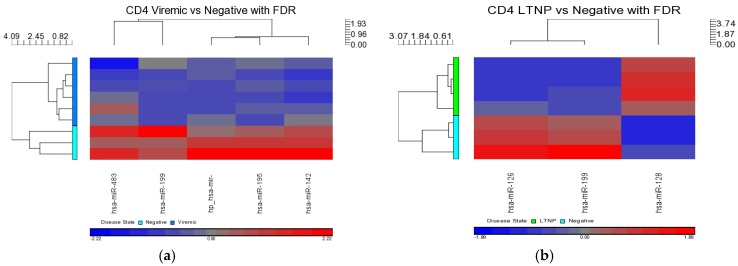
Heat maps indicating DE miRNAs in CD4+ T-cell disease group comparisons of viremic (**a**) and LTNP (**b**) compared to negative controls. FDR method was used. Viremic *n* = 6, LTNP *n* = 4, negative *n* = 3.

When HIV-infected cells were compared to uninfected cells (Figure 5) using the FDR method, miR-199a-5p was found to be downregulated in CD4+ T-cells compared to negative cells over 5-fold, whereas miR-720 was upregulated nearly 4-fold. In CD8+ T-cells, the miR-941-3-s was upregulated nearly 3-fold, where the miR-1323 was downregulated nearly 4-fold (Table 2 and Figure 5). No overlapping miRNAs between HIV+ and HIV– cells were seen, and the differential expression was unique to CD4+ and CD8+ T-cell subsets, as shown in the Venn diagram (Figure 6), suggesting that the DE miRNA are cell-specific signatures that are unique to a T-cell subset within the infected individual. This is the first evidence demonstrating cell-specific miRNA during HIV disease stages.

Apart from using the FDR method with *p* < 0.05, we also visualized the dataset using no FDR with *p* < 0.01. It is important to state that, when no FDR was applied to the analysis of differentially expressed miRNAs in infected *vs.* uninfected CD4+ and CD8+ T-cells, more miRNAs were observed, as expected. However, in this analysis, the top-ranked highly significant miRNAs that were observed in the FDR method were also fully covered in the non-FDR analysis. This dual-analysis served to ensure that statistical stringency did not occlude biologically relevant miRNAs (Table A1).

### 2.5. A Closer Examination of Differentially Expressed miRNAs in CD4+ T-Cells and Their Association with Disease Groups

#### 2.5.1. CD4+ Disease Groups *vs.* the HIV Negative Group

**Figure 7:**
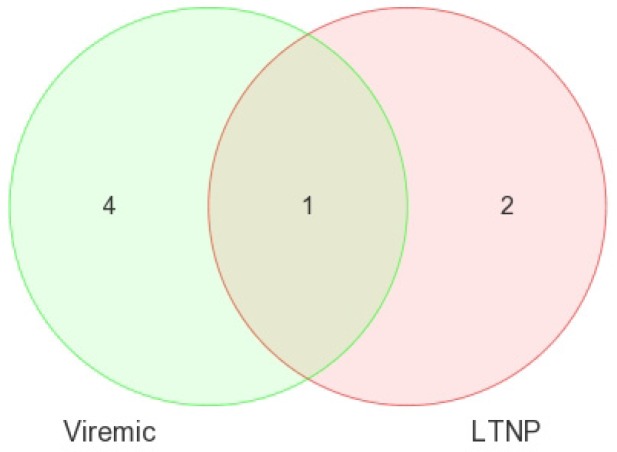
Venn diagram of DE miRNA in CD4+ T-cells when disease groups are compared to HIV– controls. FDR method was used. This diagram shows the lack of results for aviremic samples and overlapping expression of one miRNA; miR-199a-5p.

Independent inter-group comparisons were carried out for the DE miRNAs for each cell type. First, the CD4+ T-cells from unique disease groups (viremic, aviremic, and LTNPs) were individually compared against CD4+ T-cells from the HIV– controls using the FDR *p* < 0.05. Five DE miRNAs were identified in the viremic CD4+ T-cells (miR-199a-5p, miR-142-5p, miR-520f, miR-195, and miR-483-5p), and three DE miRNAs were identified in CD4+ LTNPs (miR-1995p, miR-1280, and miR-126) (Figure 6). All of these showed strong *p*-values and fold changes (Table 3). Interestingly, miR-199a-5p was found to be overlapping between these two contrasts (Figure 7).

No DE miRNAs were found in the aviremic CD4+ T-cells when compared with the HIV– controls using the FDR method. However, when the non-FDR method was used, DE miRNAs were observed in the aviremic CD4+ T-cells (Table A2), including the miRNA miR-199a-5p, which was lacking for this comparison due to statistical stringency introduced by the FDR method (Table 3). It is important to reiterate that the miRNA-199-5p is one of the most notable miRNAs, as it was unique to HIV-infected CD4+ T-cells. Viremic and LTNP comparisons demonstrated this miRNA as significant using the FDR data, but significance in the aviremic comparison appeared only to relax the statistical stringency to non-FDR with *p* < 0.01. This suggests that, although miR-199a-5p was differentially expressed in HIV infection in all three disease states, it was not expressed significantly enough to be picked up for CD4+ aviremic *vs.* HIV− comparison due to statistical stringency. Thus, taken together, the FDR (Table 3, Figure 7) and the non-FDR data (Table A2) concur in that miR-199a-5p was a signature unique to HIV+ CD4+ T-cells and may have a considerable biological significance. Interestingly, the fold changes for each contrast (CD4 HIV+ *vs.* HIV− = −5.6; viremic *vs.* negative = −6.31; LTNP *vs.* negative = −6.85; and aviremic *vs.* negative = −4.13 (non-FDR)) indicate that miR-199a-5p was downregulated in the viremic state and that its upregulation appears to be characteristic of an HIV– state in healthy individuals (Table 2).

#### 2.5.2. CD4+ T Cells Inter-Disease Group Comparisons

**Figure 8:**
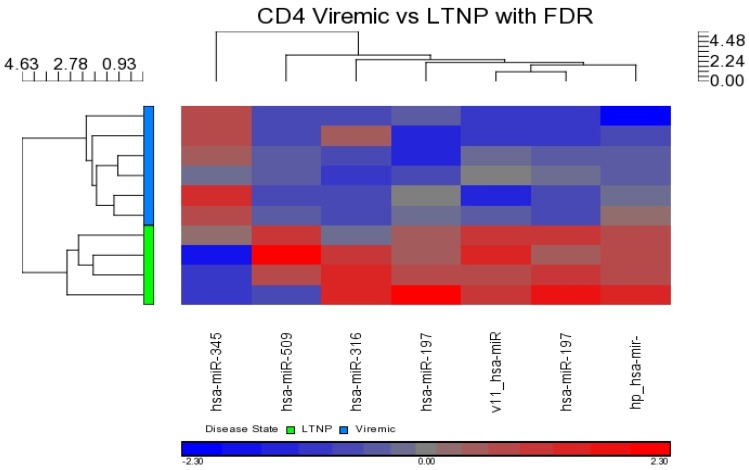
Heat map showing DE miRNAs in CD4+ viremic *vs.* LTNP disease group comparison. Viremic *n* = 6, LTNP *n* = 4.

Within the CD4+ T-cell subset, differential expression of miRNAs was intercompared between disease states. Three comparisons were examined: viremic *vs.* aviremic, viremic *vs.* LTNP, and LTNP *vs.* aviremic groups, respectively. No differential expression was observed in the viremic *vs.* aviremic or LTNP *vs.* aviremic comparisons when the FDR method was used. In the viremic *vs.* LTNP comparison, seven DE miRNAs were detected (miR-509-3p, miR-345, miR-1972, miR-1975, miR-3163, miR-768-3p, and miR-1201)—six downregulated and one upregulated. This upregulated miRNA, miR-345, demonstrated a 11.6-fold change with a *p*-value of 3.79 × 10^−5^ (Figure 8 and Table 4), but the downregulation of miRNAs during viremia was the main feature in this comparison.

When the non-FDR method with *p*-value < 0.01 was used, differential expression of a large number of miRNAs was detected for each comparison listed in Table A3.

### 2.6. A Closer Examination of Differentially Expressed miRNAs in CD8+ T-Cells and Their Association with Disease Groups

#### 2.6.1. CD8+ Disease Groups *vs.* the HIV Negative Group

Similar to the CD4+ T-cells, CD8+ T-cell samples were also examined according to their disease groups against the CD8+ T-cells from HIV– controls.

**Figure 9:**
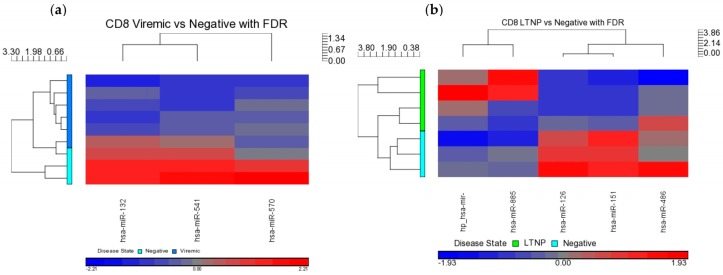
Heat maps indicating DE miRNAs in CD8+ disease group comparisons of viremic (**a**) and LTNP (**b**) compared to negative controls. Viremic *n* = 6, LTNP *n* = 4, negative *n* = 3.

Using the FDR method and *p* < 0.05, three miRNAs were found to be DE in viremic CD8+ T-cells, (miR-1323, miR-541 and miR-570), and all three showed downregulation (Table 5 and Figure 9). One miRNA (miR-1298) was specific to the aviremic group, showing 2-fold downregulation (Table 5). This is the first miRNA that is specific to CD8+ T-cells of HIV patients that control HIV through highly active antiretroviral therapy (HAART) therapy, and may have biological significance.

Further, five DE miRNAs were identified in the LTNP and HIV– controls, with miR-572, miR-126, and miR-885-3p being downregulated, and miR-151-5p and miR-486-5p being upregulated, and supported by strong *p*-values (Table 5 and Figure 9). Interestingly, several downregulated miRNAs (572, 126, and 885-3p) showed noticeably high fold changes (−15, −31, and −29 fold, respectively), and all of them were specific to the CD8+ T-cells from the LTNP group (Table 5).

**Figure 10:**
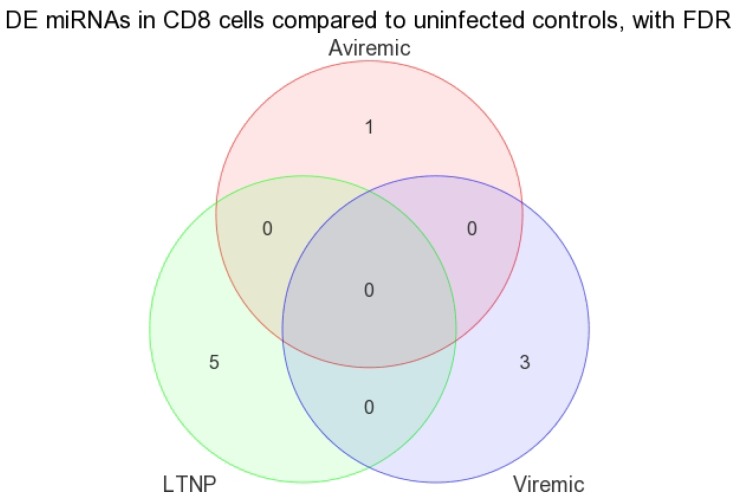
Venn diagram of DE miRNA in CD8+ T-cells when disease groups were compared to HIV– controls. FDR method with *p* < 0.05 was used. Viremic *n* = 6, aviremic *n* = 5, LTNP *n* = 4, negative *n* = 3.

Most notable was that, when all three CD8+ disease groups were intercompared using a Venn diagram, no overlapping miRNAs were found (Figure 10), suggesting the identification of all disease-state-specific miRNAs in each case were supported by strong *p*-values (Table 5). This is the first evidence of HIV disease-state-specific miRNAs based on the CD8+ T-cells and is consistent with the role of CD8+ T-cells in containing viremia. Therefore, these miRNAs may have considerable biological significance in gene regulation in CD8+ T-cells.

As expected, the non-FDR method resulted in differential expression of more miRNAs than the FDR method between disease groups based on this cell type (Table A4). Again, there was a lack of evidence for overlapping miRNA between disease states using the non-FDR method.

#### 2.6.2. CD8+ Inter-Disease Group Comparisons

Within the CD8+ subset, differential expression of miRNAs was intercompared between disease states. The three comparisons examined were viremic *vs.* aviremic, viremic *vs.* LTNP, and LTNP *vs.* aviremic. Differential expression was seen in both the viremic *vs.* LTNP and LTNP *vs.* aviremic comparisons, but not for the viremic *vs.* aviremic comparison.

**Figure 11:**
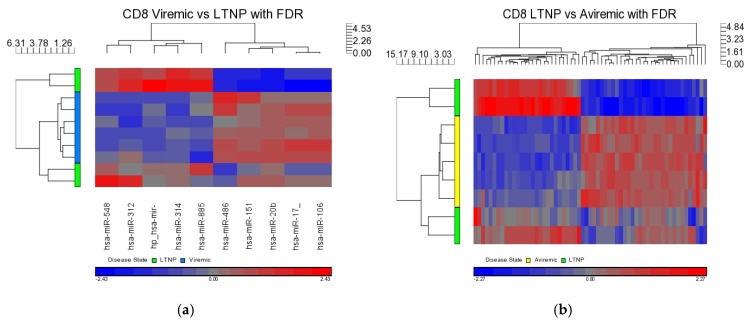
Heat maps indicating DE miRNAs in CD8+ viremic *vs.* LTNP (**a**) and LTNP *vs.* aviremic (**b**) disease group comparisons. FDR method with *p* < 0.05 was used. Viremic *n* = 6, aviremic *n* = 5, LTNP *n* = 4.

Based on the lack of differential expression seen between viremic and aviremic cells in both CD4+ and CD8+ T-cell comparisons, it is apparent that there was little separation between the viremic and aviremic states in the presence or absence of viremia while on HAART, suggesting that the same miRNAs may regulate viremic and aviremic phases of HIV infection during therapy. Differential expression in the CD8+ T-cells was observed only in viremic *vs.* LTNP and LTNP *vs.* aviremic comparisons. In the viremic *vs.* LTNP contrast, 10 DE miRNAs (5 upregulated and 5 down regulated) were identified (Table 6; Figure 11), whereas in the LTNP *vs.* aviremic contrast, 61 DE miRNAs were identified (34 were down regulated, and the remaining 27 miRNAs were upregulated), all of which were supported by strong *p*-values (Table 6 and Figure 11).

Non-FDR data was also visualized for the CD8+ T-cells, and, as expected, a large number of miRNAs were detected for each comparison (see Table A5).

### 2.7. Comparison of Differential Expression of miRNA between CD4+ and CD8+ T-Cells

**Figure 12:**
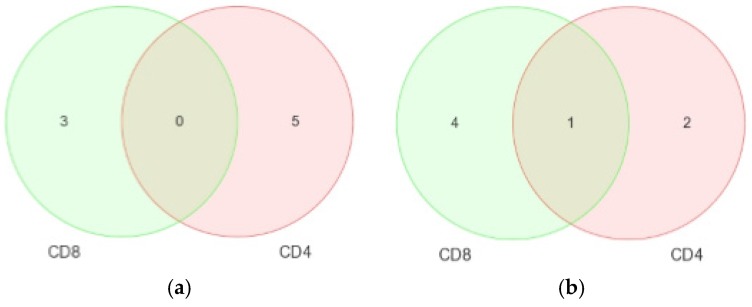
Venn diagrams showing DE miRNAs in the CD4+ and CD8+ cells of viremic (**a**) and LTNP (**b**) disease groups. FDR method with *p* < 0.05 was used.

The main reason for these comparisons was to derive specificity of CD4+ or CD8+ T-cells in predicting a disease association. To do this, simultaneous comparison of the relationship between miRNA expression CD4+ and CD8+ T-cells in different disease groups for both cell types was carried out. Table 7 shows the side-by-side comparisons, which concur with the data already stated in Table 3 and Table 5. Since few results were obtained for the comparisons involving aviremic cells, Venn diagrams were generated comparing differential expression of miRNAs in viremic (Figure 12a) and LTNP (Figure 12b**)** cells only.

For the first contrast, we found three DE miRNA for the CD4+ T-cells and five for the CD8+ T-cells during viremia. No overlapping miRNAs were seen. In contrast, for the LTNP CD4+ and CD8+ T-cells, four DE miRNAs in CD4+ T-cells and five in CD8+ T-cells during the non-progressive phase of the HIV disease were identified. The miRNA-199a-5p was found to be specific to the HIV+ CD4+ T-cells. Interestingly, miRNA-126 was the only miRNA found overlapping between CD4+ and CD8+ T-cells during the non-progressive HIV disease, which, according to the current literature, is the first miRNA possibly related to the non-progression of HIV disease (Table 7 and Figure 12b). No differential expression was seen for the aviremic comparison. This suggests a degree of similarity in the miRNA profiles of the CD4+ and CD8+ T-cells of aviremic patients undergoing HAART (Figure 12a,b).

### 2.8. Direct Comparison of DE miRNAs of the Same Disease State between CD4+ and CD8+ T-Cells

A direct comparison of CD4+ with CD8+ T-cells for the same disease group was carried out to obtain disease-stage-specific miRNAs common to both cell types. We performed comparisons between viremic CD4+ *vs.* CD8+ T-cells, aviremic CD4+ *vs.* CD8+ T-cells, LTNP CD4+ *vs.* CD8+ T-cells, and negative CD4+ *vs.* CD8+ T-cells using the FDR method to identify DE miRNAs for each disease group. Using the FDR method and an adjusted *p* < 0.05, only one miRNA was differentially expressed. This miRNA was miR-151-5p, and was differentially expressed between the CD4+ and CD8+ T-cells of the LTNP group with a fold change of 10.26 and a *p*-value of 1.95 × 10^−5^, suggesting that it is a possible biomarker of non-progressive HIV disease based on both cell types.

**Figure 13:**
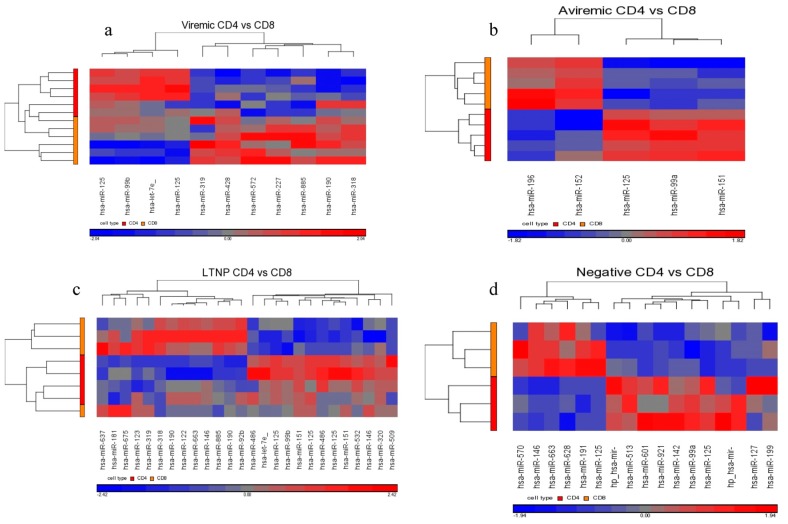
Heat map representation of miRNAs DE between CD4+ and CD8+ T-cells from disease groups viremic (**a**); aviremic (**b**); LTNP (**c**); and negative (**d**). Non-FDR method with *p* < 0.01 was used. For each cell type, viremic *n* = 6, aviremic *n* = 5, LTNP *n* = 4, and negative *n* = 3.

Since defining a disease-stage-specific miRNA based on two different T-cell types is an important biological attribute in clarifying disease and cell type association, we also opted for using the non-FDR method with *p* < 0.01. By relaxing this statistical stringency, differential expression was observed for all contrasts (Figure 13a–d and Table 8). A number of disease-stage-specific miRNAs were identified for viremic, aviremic, LTNP, and HIV– stages based on both CD4+ and CD8+ T-cells (Table 8), including the previously mentioned miRNA miR-151-5p in the LTNP comparison. Notably, even HIV– individuals showed cell-specific miRNAs, suggesting distinct miRNAs guiding cellular immunity functions of CD4+ and CD8+ T-cells during the disease-free state. This is the first evidence showing this phenomenon.

### 2.9. qRT-PCR Validation

Statistical significance of individual miRNA was used to pick two highly significant and important miRNAs to analyze by qRT-PCR. The fold changes determined for each comparison by qRT-PCR after an averaging of duplicates and calculation are shown in Table 9.

The results of qRT-PCR shown in Table 9 show consistent trends between the results of microarray analysis and qRT-PCR analysis for two miRNAs (126 and 199a-5p). Raw amplification values are presented in Appendix, Table A6. For miR-126, the significant downregulation in LTNP individuals seen in the microarray (FC-50 for CD4+ T-cells and -30 for CD8+ T-cells) was supported by qRT-PCR analysis. Large negative fold changes were identified, particularly in CD4+ T-cells, where the fold change determined was greater than −100 fold.

miRNA-199a-5p was significant and found to be specific to HIV+ CD4+ T-cells in viremic and LTNP groups using the FDR method (*p* < 0.05), but was seen also in the aviremic groups only upon relaxing the FDR (*p* < 0.01).

The results of qRT-PCR for miR-199a-5p showed a consistent trend with the results of the microarray. In the CD4+ viremic *vs.* negative analysis, downregulation (FC-2.7) observed in qRT-PCR was consistent with the trend (FC-6.31). In the CD4+ T-cells—HIV+ *vs.* HIV− and LTNP *vs.* HIV negative comparisons—the qRT-PCR results also showed consistent trends of expression with the microarray. In the HIV+ *vs.* HIV− comparison, the microarray result of −5 fold downregulation was consistent with the qPCR result of −209. Again, in the LTNP *vs.* negative CD4+ T-cells, the downregulation detected in the microarray analysis (−7 fold) was also consistent in trend seen in qRT-PCR, where the detected fold change was −50 fold.

qRT-PCR analysis was also performed for the comparison CD4+ aviremic *vs.* negative for miR-199a-5p. This comparison was significant for the microarray only in the non-FDR analysis. The fold change determined by qRT-PCR was −2.15 fold (data not shown), which is again in agreement with the trend of downregulation seen for this miRNA in CD4+ HIV-infected T-cells in the microarray.

## 3. Discussion

Along with their association with pathological and physiological pathways, host miRNAs play a significant role in many viral infections, including HIV. In this work, we describe the first direct examination of virus-mediated cellular miRNA expression manipulation in the CD4+ and CD8+ T-cell fractions taken from HIV+ patients at differing stages of HIV disease. This disease-stage stratification was primarily based on the detectable virus in the plasma of diseased individuals and the length of infection. The main rationale behind conducting a parallel analysis of CD4+ and CD8+ T-cells for miRNAome was based on the roles of these cells in HIV infection. CD4+ T-cells are the primary targets of HIV [11], whereas the CD8+ T-cells mediate antiviral control replication through cytotoxic and non-cytotoxic mechanisms. The effectiveness of this response directly correlated with prognosis [12,13]. It is evident that the HIV infection of host cells leads to an alteration of the global RNA interference machinery [14]. Although it is possible that HIV-induced changes in cellular miRNA expression results from combinatorial molecular interactions among proteins, transcripts, and genomes, the mechanisms behind these changes during host-virus interactions remain to be understood. Here, we demonstrate the first evidence for cell-, disease-stage- and HIV-specific host miRNA during HIV disease stages.

Previous studies have shown unique and variable global modifications in microRNA expressions exhibited by different host cell types and cell lines in response to HIV-1 infection [6,7,15,16,17]. These findings showed a consistent and prominent trend of downregulation of cellular miRNAs in HIV-infected individuals. For instance, in 2008, Houzet *et al.* showed a prominent trend towards downregulation of miRNA in HIV+ individuals, with 59 of 62 DE miRNAs downregulated in HIV+ PBMCs [7]. This finding is in agreement with other studies performed on cell lines [6,15], which found that when HELA cell lines are transfected with HIV-1 in a pNL4-3 vector, >43% of cellular miRNAs were downregulated, with the majority of the remainder unchanged. Our study concurs with these findings and further demonstrates that the downregulation of cellular miRNAs was systematic across both CD4+ and CD8+ T-cells and diverse disease groups. This trend of cellular miRNA downregulation was confirmed in our qRT-PCR expression trends, suggesting the functional significance of downregulation of cellular miRNAs during HIV infection and guiding the disease course.

A large body of work on miRNAs has been done on cell lines rather than primary patient cells. It is important to iterate that miRNA signatures of PBMCs from HIV+ patient blood are more than 50% discordant with those of *in vitro*-infected PBMCs [7]. This implies that a significant portion of the miRNA signature changes seen in HIV+ patients cannot be accounted for by infection alone. These changes could be attributed to *in vivo* interaction between cell types and other combinatorial interactions, which together play a functional role in determining the dysregulation of host miRNAs during HIV infection. Thus, the analysis of primary CD4+ and CD8+ T-cells we have performed from diverse HIV disease groups carry considerable functional relevance in the context of HIV infection *in vivo*.

To obtain differentially and statistically significant miRNA candidates, we employed the Benjamini–Hochberg FDR corrective testing with a *p*-value cut-off of <0.05 throughout. The Benjamini–Hochberg algorithm is designed to sort through a list of *p*-values and determine the likelihood of a false rejection of the null hypothesis (a false positive) and so “adjust” the *p*-value to control the likelihood of the false positive. Employing this algorithm provided data of greater integrity, and this is the data we have made use of in interpreting our DE dataset. Due to the stringency of this method of analysis, we failed to detect differential expression of miRNAs in some comparisons, while the DE miRNAs detected for other groups were statistically strong. To clarify any possible occlusion of biologically relevant miRNAs, we also analyzed the dataset without FDR corrective testing. To minimize false positives in the absence of FDR correction, the *p*-value cut-off was then set at <0.01. This allowed the detection of other DE miRNAs for the groups we failed to detect DE miRNAs using FDR corrective testing. Our hypothesis for using this rationale was that the statistical stringency possibly occludes biologically relevant miRNAs, as several of these miRNAs identified at *p* < 0.01 are not only of functional relevance, but are also known in the literature.

### 3.1. Signature miRNA in HIV Disease Class Prediction

Through the use of PCA, we demonstrated that the miRNA profiles of HIV+ CD4+ and CD8+ T-cells were distinct from T-cells of HIV– individuals as all HIV-infected groups (viremic, aviremic, and LTNPs) were more closely related as a group and segregated from the uninfected group. PCA also highlighted that the individual samples from the same disease stages cluster together, and the sample classes with the greater similarities were more closely associated. Despite the close relationship of all HIV+ samples, PCA could also distinguish between HIV disease sub-groups, with the LTNP, aviremic, and HIV groups showing a common miRNA signature specific to them. This is plausible, as these three groups have undetectable levels of plasma viremia. Our results can be likened to the results shown by Witwer *et al.* on PBMCs, where the miRNA signatures of LTNP HIV+ individuals with low viral load and good CD4+ T-cell counts showed greater similarities to uninfected controls than HAART-treated HIV+ patients.

In support of these findings, several previous papers have suggested that a broad statistical analysis of the miRNAs that are differentially expressed in HIV+ individuals distinguish patients according to their CD4+ T-cell counts, viremia, or HIV exposure level [7,9,15]. In 2008, Houzet *et al.* showed that HIV+ patients could be categorized into disease groups based on DE miRNAs present in the PBMC fraction of their blood. Further, the miRNA signature of CD4+ T-cells from multiply exposed uninfected (MEU) individuals can distinguish them from therapy-naïve infected CD4+ T-cells [15]. Bignami *et al.* also showed that miRNA signatures of LTNPs, MEUs, and therapy-naïve HIV+ patient CD4+ T-cells cluster based on their exposure level and disease state [15]. As mentioned, Witwer *et al.* showed that, through examination of DE miRNAs, LTNPs with low CD4+ T-cell counts and high plasma viremia correlate more closely with HIV+ viremic individuals, and those with high CD4+ T-cell counts correlate with HIV− controls [9]. Thus, the characteristics of an miRNA signature allowed accurate assignment of samples into disease states using a variety of predictive programs [9], which is what our analysis has shown at both cell-type and disease-stage levels.

### 3.2. DE miRNAs in CD8+ T-Cell Predict HIV Disease Stage Better than CD4+ T-Cells

When DE miRNAs were analyzed in the CD8+ T-cells of HIV+ individuals from each of the disease groups (viremic, aviremic, and LTNP), it was shown that some miRNAs were highly specific for these disease groups when compared with negative controls. Namely, miR-1323, -541, and -570 were associated with the CD8+ viremic group, miR-1298 was associated with the CD8+ aviremic group, and the miRNA-572, -126, 151-5p, -486-5p, and -885-3p with the CD8+ LTNP group. This is the first direct evidence of HIV disease-stage-specific miRNAs in CD8+ T-cells, which is consistent with the role of CD8+ T-cells in containing viremia. A clear segregation of DE miRNA in each comparison demonstrates that the CD8+ T-cells are better suited to predicting HIV disease stage and cell-specific miRNAs than their CD4+ T-cell counterparts.

### 3.3. Unique miRNAs in Non-Progressive HIV Disease

Our analysis performed in CD4+ and CD8+ T-cell types suggests that miRNAs-199a-5p, -1280, and -126 were differentially expressed in the CD4+ T-cells of LTNPs when compared to negative controls, and the miRNAs-151-5p, -486-5p, -572, -885-3p, and -126 were differentially expressed in the CD8+ T-cells of LTNPs when compared to negative controls. MiR-126 was differentially expressed in both CD4+ and CD8+ T-cells in the LTNP disease state. Consistent expression trends between microarray and qRT-PCR for miR-126 suggests that this miRNA is significantly downregulated in both the CD4+ and CD8+ T-cells of LTNPs, suggesting that it is a possible biomarker for non-progressive HIV disease. This, to our knowledge, is the first report of a miRNA that correlates with the LTNP disease state and may be involved in the regulation of cellular immunity and possibly the natural control of HIV in LTNPs.

MiR-199a-5p was differentially expressed in HIV+ CD4+ T-cells belonging to all HIV+ groups analyzed (viremic, aviremic, and LTNPs). Stronger downregulation was observed in LTNP *vs.* negative disease group than in either the viremic or aviremic CD4+ T-cell comparisons against HIV− individuals. Thus, it is likely that miR-199a-5p plays a role in predicting HIV+ status based on CD4+ T-cells. We hypothesize that, although it is expressed and downregulated in the CD4+ T-cells of all HIV+ groups, the degree of downregulation of miR199a-5p in different groups imparts susceptibility or protection, as evident from the CD4+ T-cells from the LTNPs that showed the highest degree of downmodulation of miRNA199-5p, which may play a protective role against the virus. Supporting this conclusion, it has previously been shown that miR-199a (along with miRNAs-143, -303-3p, and -335) is differentially expressed in the PBMCs of class 1 (HIV+, high CD4+ T-cell count, low viral load) individuals, which agrees with our work with miRNA 199a showing the strongest downregulation in the LTNPs group. Our analysis using three phenotypically different HIV+ groups clarifies and unambiguously demonstrates a clear association of miR-199a with HIV positivity based uniquely on CD4+ T-cells, a trend which was further confirmed by qRT-PCR analysis, implicating miR-199a to be an HIV-specific miRNA.

About other differentially expressed miRNAs, miRNA-155 in HIV+ CD4+ T-cells was recently observed to distinguish LTNPs from therapy-naive HIV+ patients [15]. They suggest that high miR-155 in CD4+ T-cells may correlate with HIV-1 pathogenesis. Our analysis showed similar trends to those of studies by Bignami *et al.* and Witwer *et al*.

### 3.4. The miR-125 Family Is Important in the Maintenance of Latency and Aviremia

The 3′ ends of HIV-1 mRNAs (Nef) are targeted by cellular miR-125b (along with miR-28, miR-150, miR-223, and miR-382), all of which are enriched in resting CD4+ T-cells compared to active CD4+ T-cells [8]. These miRNAs have inhibitory effects and enforce viral latency in resting HIV+ CD4+ lymphocytes by reducing protein translation and viral production [8]. Moreover, miR-125b has been identified as one of the constitutive miRNA signatures of naïve CD4+ T-cells [18]. MiR-125a-3p, along with miR-23 and miR-155, is shown to be upregulated in HIV+ therapy-naïve CD4+ T-cells, normal in LTNP CD4+ T-cells, and downregulated in the CD4+ T-cells of MEU samples [15]. A recent study involving PBMC miRNA profiling [9] did not find miR-125b correlating with replication control and latency; however, this study involved only the whole PBMCs and not the individual cell subsets [18,19], which may harbor unique miRNA characteristics shown in our study.

Interestingly, our results show that the family of miR-125 was consistently upregulated in the comparison of diseased CD4+ T- and CD8+ T-cells using a non-FDR statistical analysis. This upregulation was markedly higher in the CD4+ T-cells of LTNPs than those of viremic or aviremic individuals, followed by its upregulation in aviremic individuals, suggesting a possible role for this family of miRNAs in aviremia and natural control and possibly viral latency. Our results agree in part with the findings by Huang *et al.,* and we concur that miR-125 family members are potential candidates associated with latency in CD4+ T-cells [8]. More work is needed to prove the biological relevance of this miRNA. It is important to iterate that this differential expression was only achieved when less stringent corrective testing was applied. Nonetheless, the fold changes and *p*-values of miRNA candidates with the disease stage added reliability and confidence to these conclusions.

### 3.5. Possible Role of miR-29 Family in Viral Replication and Management of Viremia during HAART Treatment

miR-29 targets the viral Nef 3′UTR and downregulates Nef protein expression and interferes with HIV-1 replication, thereby inhibiting viral production and infectivity in HIV-infected T lymphocytes [7]. Low levels of miR-29b are associated with disease progression and viremia [9,10], whereas high expression of miR-29b positively correlates with CD4+ T-cell count. Furthermore, miR-29a specifically targets the HIV-1 3′UTR [10,20,21]. This implies a role for miR-29 members in prohibiting viremia and disease progression [9]. Recently, it has been discovered that miR-29 suppresses HIV-1 replication and binds directly to HIV-1 Nef mRNAs [22]. An indirect role for miR-29 has also been suggested in the modulation of cyclins in HIV infection [23].

Although our results did not immediately suggest a role for miR-29 in managing viremia and disease progression, it became clear upon examination that miR-29 was indeed upregulated in aviremic CD4+ T-cells, when compared to both viremic and LTNP CD4+ T-cells. Although it is peculiar that the miR-29s did not show association with the LTNP disease state, this is not unexpected. The biggest difference between HIV+ aviremic and LTNP individuals is the preservation of immune system strength and natural control of HIV in LTNP individuals, where aviremic individuals require HAART to control HIV replication. It is likely that miRNAs may have some role in maintaining below detectable levels of plasma viremia while on HAART therapy. On another note, it is important to iterate that we also found miR-1298 specific to the CD8+ aviremic group (with respect to negative control), showing 2-fold downregulation. This was obtained using the FDR corrective testing, and we believe it is the first miRNA specific to CD8+ T-cells of HIV patients that may be involved in control HIV during HAART therapy and may offer some biological significance. Thus, these two miRNAs may have some functional relevance during HAART therapy, and whether they come into play when therapy is effective in controlling plasma viremia remains unknown. Nonetheless, the two miRNAs in some ways predict the efficacy of HAART and should be explored in the context.

Similarly, in aviremic CD8+ T-cells, miR-29b/c were upregulated when compared to negative controls, whereas the miR-29a/b/c were upregulated in aviremic individuals when compared to LTNPs. Taken together, these data suggest that the expression of the cellular miR-29 family is triggered by viral replication and implies a potential role for miR-29 family members in both CD4+ and CD8+ T-cell subsets in HIV infection, although perhaps not in the management of viremia in the LTNP group. Determining whether miR-1298 and the miR-29 family have some functional synergy between them requires future functional validation.

### 3.6. miRNA-17/20 Are Downregulated in CD8+ T Cells in LTNPs: A Tentative Link to Their Role in Viral Replication

miR-17/20 target 3′UTR of PCAF acetyltransferase and are known to indirectly inhibit HIV infection [7,8]. It has been shown that the transfection of miR-17-5p and -20 into the PBMCs of HIV-1 infected donors leads to reduced HIV-1 production, and that transfection of antisense inhibitor enhances PCAF expression and HIV-1 production [24]. PCAF1 is an important cofactor for tat in HIV-1 gene expression and has potential sites for these miRNAs in its 3′UTR. Recently, miR-17a has been associated with enhanced viral production and predicted to bind cellular factors that indirectly influence HIV-1 replication [22].

We found that miR-17 and miR-20a were both downregulated in the CD8+ T-cells of LTNPs when compared directly with the CD8+ T-cells from aviremic patients. MiR-17 was downregulated in CD8+ T-cells from the LTNPs when compared against the viremic patients. This upregulation of miRNAs could be related to the inhibition of HIV-1 in a more severe disease state (aviremic on HAART compared to LTNPs), rather than the downregulation of these miRNAs by HIV.

As can be noted in several sections of the discussion, the correlation of DE miRNAs observed in our study could only be compared with other studies when non-FDR corrective testing was used. Thus, it is important to reiterate that, since we performed a bimodal analysis of our dataset using FDR (*p* < 0.05) and non-FDR (*p* < 0.01) statistical analysis, we were able to observe both the strongly DE miRNAs responsible for clear segregation of disease groups using FDR analysis, as well as the gentler trends of miRNA expression between disease classes and cell types in the non-FDR analysis. Our observations regarding the two datasets raise two possibilities: that either most studies in the miRNA field are not as stringently controlled as our primary FDR analysis, or that the stringency we have employed in deriving significantly DE miRNA is too strict. If this is the case, high stringency analysis may occlude biologically significant miRNA candidates, as was apparent when functionally important miRNA deemed important in the literature were confirmed and supported by our non-FDR dataset.

In summary, the findings presented herein provide evidence that HIV infection plays a significant role in altering cellular miRNA expression and, conversely, that cell-specific miRNA expression may have implications for the control of HIV infection. We report the findings of a variety of miRNAs that are differentially expressed in a cell-, disease-stage- and HIV-infection-specific manner, and offer support to the findings of several other studies. This is the first evidence showing tight miRNA regulation during HIV disease in different cell types and disease stages, suggesting an essential role for miRNA-guided cell phenotype in HIV pathogenesis and the development of AIDS. A unique aspect of these findings is the evidence for miRNAs that are involved in HIV resistance, as seen in non-progression. Further study of the roles of these miRNAs, their cognate mRNAs, and the reason for their dysregulation in HIV infection may shed light on the mechanisms controlling the progression or non-progression of HIV disease, and open up a pathway to a new line of therapeutics for the effective treatment and control of HIV.

## 4. Materials and Methods

### 4.1. Fresh PBMC Isolation from Whole Blood

Up to 10 mL of freshly collected whole blood in EDTA was diluted to 35 mL with DPBS (Dulbecco’s phosphate buffered saline, Lonza BioWhittaker, Walkersville, MD, USA) and layered carefully onto 15 mL of Ficoll (Ficoll-Paque PLUS, 1.077 ± 0.001 g/mL, GE Healthcare, Uppsala, Sweden) in 50-mL tubes, without disrupting the Ficoll border. Separation of the PBMC buffy coat was achieved via centrifugation at 1800 rpm for 20 min at room temperature. The buffy coat containing peripheral blood mononuclear cells (PBMCs) was extracted to another 50-mL tube via transfer pipette. The separated PBMCs were washed twice with DPBS by centrifugation at 1500 rpm for 7 min at 4 °C, then once in 10 mL of cold (4 °C) magnet activated cell sorting method (MACS) buffer (DPBS pH 7.2, 2 mM EDTA (Amresco, Sydney, Australia), 0.5% FBS (Fetal Bovine Serum, SAFC Biosciences, Lenexa, KS, USA) via centrifugation at 1200 rpm for 10 min at 4 °C. The supernatant was aspirated retaining the PBMC pellet.

### 4.2. Processing of Cryopreserved PBMCs

PBMCs from the LTNPs used in this study were previously cryopreserved. These cells frozen in liquid nitrogen were raised to −80 °C for one day before revival. Cells were slowly thawed on ice and were transferred using a transfer pipette into 15-mL tubes containing 5 mL of RPMI 1640 (Lonza BioWhittaker) 10% FBS in 15-mL tubes. The cell suspension was centrifuged for 10 min at 1000 rpm at room temperature to pellet the revived cells. The supernatant was aspirated and decanted retaining the clean cell pellet in preparation for the CD4+ and CD8+ cell separation.

### 4.3. Macs Column Separation of CD4+ and CD8+ T Cells from Whole PBMC

The PBMCs were centrifuged at 1000g to remove cell debris. The cell pellet was further washed twice with phosphate buffered saline (PBS) to remove excess antigen. After aspirating all of the PBS, the cell pellet was further washed with 10ml of MACS buffer (50 mL PBS + 250 µL of Fetal Bovine Serum (FBS) + 200 µL of 2 mM EDTA) by centrifuging at 300 *g* for 10 min at 4 °C. Cells were washed with PBS, and MACS^®^ Column Technology was used for separating PBMCs into different cell subsets. Using MACS^®^ MicroBeads, CD4^+^ helper T-cells and CD8^+^ cytotoxic T-cells were extracted according to the manufacturer’s specifications using the positive selection MACS, Miltenyi Biotech (Marburg, Germany). Flow cytometric verification of cell purity of CD4+ and CD8+ T-cells was evaluated using flow cytometry, as previously described by us (Potter *et al.*, 2006).

### 4.4. RNA Extraction and miRNA Enrichment

Both total RNA and miRNA were extracted from the brain cortex using the miRNeasy Mini Kit (Qiagen Pty Ltd., Clifton Hill, Victoria, Australia) with an integrated step of on-column DNase digestion as per the manufacturer’s protocol for the kit.

### 4.5. RNA Quality Check and Quantification

RNA quality was checked by RNA micro-electrophoresis on a 2100 Bioanalyzer apparatus (Agilent Technologies, Oxford, UK) and by using the RNA 6000 Nano Labchip Technology Kit. All reagents were equilibrated to room temperature for 30 min before use, and the quantity and quality of RNA were assessed based on the RNA integration numbers (RIN) for each sample. All RNA samples used in q-PCR and miRNA analysis had an RIN of >5, and anything <5 was deemed inappropriate for the miRNA chip, but were considered for the q-PCR.

### 4.6. FlashTag Biotin HSR RNA Labeling and Affymetrix GeneChip miRNA Arrays

MicroRNA was prepared for Affymetrix GeneChip arrays using the FlashTag^®^ Biotin HSR RNA Labeling Kit for Affymetrix^®^ GeneChip^®^ miRNA Arrays (Genisphere, Hatfield, PA, USA). miRNA array preparation was conducted according to instructions provided by Genisphere for the Affymetrix GeneChip miRNA array procedure (FlashTag Biotin HSR RNA Labeling Kit for Affymetrix GeneChip miRNA Arrays), including the use of reagents from this kit (vials numbered). Hybridization of prepared samples, incubation, washing, staining, and scanning was completed by the sample submission service at the Ramaciotti Center for Gene Function Analysis, University of New South Wales, Sydney.

### 4.7. Analysis

#### 4.7.1. Sample Classification and Criteria

Patients were classed into disease groups based on their HIV plasma VL determined by M3 Prep/Cobas TaqMan HIV-Version 2 kit (ROCHE, Inc., Pleasanton, CA, USA) to test HIV plasma VL, at the Westmead Hospital ICPMR (Institute of Clinical Pathology and Medical Research). Diverse HIV disease groups were analyzed, including HAART-experienced viremic (VL above 40 copies/mL plasma) and aviremic (below detectable levels of plasma viremia) patients. An LTNP group, comprising of HIV+ therapy-naïve individuals who showed high CD4+ and CD8+ T-cell counts and below detectable plasma viremia in the absence of HAART, was also used. All comparisons between disease groups were made against HIV– healthy individuals. Thus, the main criterion for analysis was the stratification of patients into viremic, aviremic, LTNP, and HIV– healthy groups, with the aim of defining disease-stage-specific and cell-specific (CD4+ and CD8+ T-cells) miRNA.

#### 4.7.2. Normalization, QC, and PCA Analysis

The Analysis was performed in the Partek Genomics Suite using inbuilt data normalization for Affymetrix CEL files (consisting of background correction, quantile normalization, Log_2_ transformation, and median polish summarization of probesets). The quality of the miRNA dataset and its integrity was assessed using PCA. PCA is a mathematical operation involving applied linear algebra, which transforms datasets into a new coordinate system such that the most variable components of the dataset are presented on axes where each component is independent of the last. This allows a complex dataset to be simplified and visualized by its most important gradients. In turn, the analysis also allows the determination of which factors are the most important in distinguishing one cluster from another. PCA plots were generated, to first test the integrity of infected samples in comparison to uninfected samples, and then PCA was used to inter-compare the disease states for each cell type and the cell types for each disease state (Figure 1, Figure 2 and Figure 3). Frequency histograms were also generated for both the CD4+ and CD8+ T-cell subsets, showing the effectiveness of normalization applied by the Partek Genomics Suite and concordance of the datasets (Figure 3).

#### 4.7.3. Statistical Analysis

Samples were analyzed using the Partek Genomics Suite software, applying two forms of corrective and statistical testing. Primarily, the analysis of differential expression was completed using *t*-tests followed by Benjmini–Hochberg FDR corrective testing. The Benjamini–Hochberg algorithm sorts a list of *p*-values in ascending order and determines how likely value is to be a false rejection of the null hypothesis (a false positive) at each point in the list. In this way, it develops a list of corrected or adjusted *p*-values for the detected differentially expressed miRNAs. A fold change of 2 (up- or downregulation) was also applied to deem any miRNA to be biologically and statistically significant. Although not a part of the result section, data were also processed without FDR corrective testing to see the difference between two datasets. This was to determine whether the statistical manipulation of the data using the FDR method occluded biologically or functionally significant miRNAs. For non-FDR data, a stricter *p* < 0.01 significance threshold was applied for significant changes, along with the fold change of 2, to ensure biological significance. This method will herein be referred to as the non-FDR method and is used as a comparative set of data for the FDR results.

Notably, even though non-FDR testing showed a higher number of DE miRNAs than FDR-controlled corrective testing, all microRNAs deemed significant using strict FDR corrective testing were also picked up as highly significant miRNAs in the less stringent, non-FDR method, confirming the reliability of FDR corrective testing.

Comparisons of miRNA expression were made in an organized and structured manner. First, differential expression of miRNAs between HIV+ and HIV– individuals was considered. Then, comparisons were made between CD4+ and CD8+ T-cells before examining the differential expression between disease states within CD4+ and CD8+ T-cells. Lastly, results observed in independent CD4+ and CD8+ T-cell analyses were inter-compared. Analyses included clustering, the generation of heat maps, and intergroup comparisons through the generation of Venn diagrams.

#### 4.7.4. Quantitative Real-Time PCR Validation of Selected and Significant DE miRNAs

Two miRNAs of significance and importance were selectively corroborated using quantitative real-time PCR (qRT-PCR) based on their adjusted *p*-values, fold changes, and differential expression. cDNA was generated from RNA samples using the Qiagen miScript Reverse Transcription Kit (Qiagen, Sydney, Australia). A master mix containing 160 µL of buffer RT and 40 µL of miScript reverse transcriptase mix was produced, and 5 µL was added to each sample of 250-ng RNA. The samples were made with RNase free water (up to 20 µL) and incubated at 37 °C for 60 min, and then 95 °C for 5 min to inactivate the RT enzyme using the Hybaid Thermal Cycler for PCR. Expression levels of two differentially expressed miRNAs (hsa-miR-199a and miR-126) were validated by qRT-PCR using the Qiagen miScript PCR system (Qiagen, Valencia, CA, USA). Master mixes containing SYBR green PCR master-mix (10 µL/assay), miScript universal primer (2 µL/assay), and RNase-free water (5 µL/assay) was produced for each specific primer. Unique miScript primers specific for the miRNAs of interest were added (2 µL/assay). Data was analyzed using Microsoft Excel. RNU6B control was used to normalize data. All the experiments were performed in duplicate, and relative expression levels of these miRNAs were determined by the ΔΔ−C_T_ method (see Table 9 for qRTPCR results).

## 5. Conclusions

MicroRNAs are the master regulators of gene expression; therefore, this analysis of differentially expressed miRNA in CD4+ and CD8+ T-cells at different stages of HIV infection provides highly valuable data on how these two T-cell subsets interact during HIV infection. Our analysis provides data on how miRNAs may guide disease progression: leading to gradual disruption and decline of the immune system in viremic individuals or providing possible resistance against HIV infection, as seen in LTNPs.

Determining the role of differentially expressed miRNAs in HIV infection is essential. We believe that an understanding of the dysregulated miRNAs in HIV infection will prove crucial for the development of miRNA-based therapies or the targeting of cellular miRNA as treatment for HIV infection. Before this is possible, a clear understanding of miRNA gene targets is needed. This is a challenge, as one miRNA can target thousands of genes, and HIV infection of cells can induce large changes in gene expression and overall cell state, meaning that the relationships between miRNAs and mRNAs in HIV infection may be very complex. As such, more work is needed to map precisely the mRNA and miRNA interactions that guide HIV pathogenesis. Such analysis will not only allow the development of new strategies for HIV control but may also lead to the identification of miRNA candidates that can be used as a new generation of therapeutic targets to control HIV. For example, a miR-122 agonist, miravirsen, is the first miRNA-targeted drug to be developed for the treatment of viral accumulation in hepatocytes during HCV infection [25].

With an increase of research in the area of miRNA expression profiling and functional analysis, several confounding factors have emerged. Currently, a variety of papers in this area all used different cell types, different definitions of HIV disease states, different methods of analysis and statistical evaluation, and diverse microarray platforms [7,8,9,15,21,23]. With all these variances in methods used by different studies, it is no surprise that the majority of research in this area is not currently in agreement. A consensus on miRNAs that is relevant in HIV disease will provide a durable framework for future work involving functional assays and miRNA/mRNA interaction analysis. Further, these clarifications will provide a clear view to developing new generation therapeutics to treat HIV disease. What is significant is that these analyses show miRNA that is specific to the natural control of HIV disease in LTNPs, which may be of vital importance in designing a new generation of prognostics and diagnostics for HIV disease.

## Figures and Tables

**Table 1:** Clinical profiles of the study patients, and RIN.

Patient	Sex	Plasma Viral Load	Disease Stage	Cell Type	RIN
VIR1	M	42,200	Viremic	CD4+	9.6
				CD8+	9.7
VIR2	M	58	Viremic	CD4+	9.5
				CD8+	9.6
VIR3	M	226	Viremic	CD4+	9.5
				CD8+	9.7
VIR4	M	5240	Viremic	CD4+	9.5
				CD8+	9.7
VIR5	M	90,300	Viremic	CD4+	9.1
				CD8+	9.3
VIR6	M	112,000	Viremic	CD4+	9.7
				CD8+	9.7
AVIR1	M	BDL	Aviremic	CD4+	9.6
				CD8+	9.4
AVIR2	M	BDL	Aviremic	CD4+	9.8
				CD8+	9.9
AVIR3	M	BDL	Aviremic	CD4+	10
				CD8+	10
AVIR4	F	BDL	Aviremic	CD4+	9.6
				CD8+	9.9
AVIR5	M	BDL	Aviremic	CD4+	9.7
				CD8+	9.6
LTNP1	M	BDL	LTNP	CD4+	9.7
				CD8+	9.8
LTNP2	M	BDL	LTNP	CD4+	8.8
				CD8+	9.8
LTNP3	M	BDL	LTNP	CD4+	8.6
				CD8+	8.1
LTNP4	M	BDL	LTNP	CD4+	10
				CD8+	8.9
HD1	M	-	Negative	CD4+	9.7
				CD8+	9.8
HD2	M	-	Negative	CD4+	10
				CD8+	9.7
HD3	F	-	Negative	CD4+	9.6
				CD8+	9.2

RIN = RNA Integrity Number. Plasma HIV viral load is in per mL plasma. BDL = Below Detectable Levels of plasma HIV as seen in therapy-naive long-term non-progressors (LTNPs) and the therapy-experienced aviremic group (AVIR). VIR = HIV-infected patients with detectable viremia while on highly active antiretroviral therapy (HAART). HD = HIV-healthy donors.

**Table 2:** DE miRNAs in HIV infected CD4+ and CD8+ T-cells using the false discovery rate (FDR) method.

Cell Type	miRNA	Adjusted *p*-Value	Fold Change
**CD4+**	miR-199a-5p	1.22 × 10^−6^	−5.6
	miR-720	6.61 × 10^−5^	3.69
**CD8+**	miR-941-3-s	5.83 × 10^−5^	2.77
	miR-1323	9.14 × 10^−5^	−3.72

Negative sign (−) signifies downregulation, whereas no sign signifies upregulation.

**Table 3:** DE miRNAs in CD4+ T-cell disease groups compared to HIV– controls.

Disease State Compared	miRNA	Adjusted *p*-Value	Fold Change
**Viremic**	miR-199a-5p	4.61 × 10^−6^	−6.31
	miR-142-5p	1.49 × 10^−4^	−3.7
	miR-520f	1.02 × 10^−4^	−2.96
	miR-195	8.72 × 10^−5^	−6.44
	miR-483-5p	7.50 × 10^−5^	−4.28
**Aviremic**	no results		
**LTNP**	miR-199a-5p	7.66 × 10^−6^	−6.85
	miR-1280	9.42 × 10^−6^	3.71
	miR-126	1.78 × 10^−6^	−51.73

Viremic *n* = 6, aviremic *n* = 6 LTNP *n* = 4, negative *n* = 3. FDR method was used. Negative fold change indicates downregulation in disease state compared to negative. Positive fold change indicates upregulation in disease state compared to negative.

**Table 4:** DE miRNAs in CD4+ T-cells derived through inter-disease group comparisons.

Comparison	miRNA	*p*-Value	Fold Change
**Viremic** *vs.* **Aviremic**	No DE miRNA		
**Viremic** *vs.* **LTNP**	miR-509-3p	7.77 × 10^−5^	−7.57
	miR-345	3.79 × 10^−5^	11.66
	miR-1972	5.31 × 10^−5^	−7.9
	miR-1975	2.03 × 10^−5^	−2.62
	miR-3163	1.10 × 10^−4^	−3.8
	miR-768-3p	9.30 × 10^−6^	−2.06
	mir-1201_s	8.31 × 10^−5^	−2.14
**LTNP** *vs.* **Aviremic**	No DE miRNA		

FDR method, *p* < 0.05. No sign indicates upregulation in the former disease state compared to the latter, whereas negative sign (−) indicates downregulation in the former disease state compared to the latter.

**Table 5:** DE miRNAs in CD8+ T-cell disease groups compared to HIV– controls.

Disease State Compared	miRNA	Adjusted *p*-Value	Fold Change	
**Viremic**	miR-1323	0.0002	−4.23	
	miR-541	0.0001	−3.95	
	miR-570	8.79 × 10^−6^	−2.18	
**Aviremic**	miR-1298	2.42 × 10^−5^	−2.02	
**LTNP**	miR-572	7.06 × 10^−6^	−14.86	
	miR-126	5.46 × 10^−6^	−31.25	
	miR-151-5p	1.41 × 10^−5^	2.35	
	miR-486-5p	7.96 × 10^−5^	6.77	
	miR-885-3p	1.89 × 10^−5^	−29.22	

FDR method with *p* < 0.05 was used. Viremic *n* = 6, aviremic *n* = 5, LTNP *n* = 4, negative *n* = 3. No sign indicates upregulation and negative sign (−) indicates downregulation in the disease state compared to negative controls.

**Table 6:** DE miRNAs in CD8+ T-cells when disease groups are intercompared.

Comparison	miRNA	*p*-Value	Fold Change	Disease State	miRNA	*p*-Value	Fold Change
**Viremic** *vs.* **Aviremic**	No Results			**LTNP** *vs.* **Aviremic (cont’d)**	miR-17	7.47 × 10^-5^	−2.49
**Viremic** *vs.* **LTNP**	miR-151-5p	2.22 × 10^−5^	8.22		miR-20a	4.10 × 10^−5^	−5.1
	miR-486-5p	4.53 × 10^−6^	19.01		Let-7d *	6.75 × 10^−4^	−2.23
	miR-885-3p	2.17 × 10^−5^	−5.95		miR-1268	4.17 × 10^−5^	4.28
	miR-3141	4.88 × 10^−5^	−2.26		miR-548u	1.23 × 10^−3^	2.05
	miR-20b	1.60 × 10^−4^	7.5		miR-199b-3p	4.34 × 10^−4^	−5.46
	miR-106a	4.98 × 10^−5^	2.44		miR-3126-3p	2.70 × 10^−4^	2.14
	miR-17	4.70 × 10^−5^	2.48		miR-502-3p	6.25 × 10^−4^	−3.71
	miR-548u	1.79 × 10^−4^	−2.3		miR-326	8.02 × 10^−4^	2.09
	miR-3126-3p	1.29 × 10^−4^	−2.18		miR-1289-2	4.14 × 10^−5^	2.48
	mir-1289-2	7.24 × 10^−5^	−2.31		miR-1207-5p	9.97 × 10^−4^	2.41
**LTNP** *vs.* **Aviremic**	miR-1909	1.40 × 10^−3^	5.57		miR-126	9.90 × 10^−4^	−8.34
	miR-151-5p	2.08 × 10^−5^	−9.03		miR-145	1.30 × 10^−3^	−16.72
	miR-1469	6.34 × 10^−4^	3.35		miR-494	5.62 × 10^−4^	4.33
	miR-663	8.00 × 10^−4^	4.18		miR-30d	4.93 × 10^−4^	−4.66
	miR-3185	7.88 × 10^−4^	6.92		miR-27a	5.93 × 10^−4^	−5.92
	miR-1908	7.06 × 10^−4^	4.31		miR-31	2.45 × 10^−4^	−11.48
	miR-151-3p	6.66 × 10^−4^	−6.34		miR-26a	7.81 × 10^−4^	−2.21
	miR-762	9.57 × 10^−5^	5.5		miR-30b	5.32 × 10^−4^	−12.55
	miR-3196	2.49 × 10^−4^	4.25		miR-19b	3.54 × 10^−4^	−7.8
	miR-1228 *	9.00 × 10^−5^	6.2		miR-4270	6.97 × 10^−4^	2.68
	miR-4281	1.11 × 10^−4^	4.14		miR-1975	6.73 × 10^−6^	2.95
	miR-92b *	1.20 × 10^−3^	4.37		miR-769-5p	8.46 × 10^−4^	−6.87
	miR-638	4.71 × 10^−5^	4.38		miR-28-3p	1.72 × 10^−3^	−2.25
	miR-1915	1.25 × 10^−4^	4.71		miR-629	1.15 × 10^−3^	−12.53
	miR-486-5p	1.90 × 10^−5^	−16.05		miR-106b	3.94 × 10^−4^	−6.18
	miR-885-3p	5.09 × 10^−5^	5.7		miR-30e *	1.51 × 10^−3^	−3.78
	miR-2861	8.69 × 10^−5^	5.53		miR-1979	1.32 × 10^−4^	7.32
	miR-3141	6.97 × 10^−6^	2.65		miR-30c	4.30 × 10^−4^	−2.56
	miR-146b-5p	4.17 × 10^−4^	−7.07		miR-30e	9.59 × 10^−4^	−9.65
	miR-149 *	4.05 × 10^−5^	7.49		miR-194	1.50 × 10^−3^	−7.99
	miR-532-5p	1.05 × 10^−3^	−3.86		miR-28-5p	8.44 × 10^−4^	−4.92
	miR-572	1.72 × 10^−3^	4.58		miR-23b	3.02 × 10^−4^	−2.5
	miR-1910	1.50 × 10^−3^	3.53		miR-25	6.61 × 10^−4^	−2.31
	miR-20b	1.34 × 10^−4^	−8.38		let-7g	2.73 × 10^−4^	−2.22
	miR-106a	1.16 × 10^−4^	−2.38		miR-182	1.62 × 10^−3^	−2.35

* FDR method. Viremic *n* = 6, aviremic *n* = 5, LTNP *n* = 4.

**Table 7:** DE miRNAs in viremic, aviremic, and LTNP cells, both CD4+ and CD8+T-cells.

CD4			CD8		
miRNA	*p*-Value	Fold Change	miRNA	*p*-Value	Fold Change
**Viremic** *vs.* **Negative**					
miR-199a-5p	4.61 × 10^−6^	−6.31	miR-1323	1.72 × 10^−4^	−4.23
miR-142-5p	1.49 × 10^−4^	−3.7	miR-570	1.27 × 10^−4^	−3.95
hp_mir-520f	1.02 × 10^−4^	−2.96	miR-541	8.79 × 10^−6^	−2.18
miR-195	8.72 × 10^−5^	−6.44	-	-	-
miR-483-5p	7.50 × 10^−5^	−4.28	-	-	-
**Aviremic** *vs.* **Negative**					
N/A			hp_mir-1298	2.42 × 10^−5^	−2.02
**LTNP** *vs.* **Negative**					
miR-199a-5p	7.66 × 10^−6^	−6.85	miR-151-5p	7.06 × 10^−6^	−14.86
miR-1280	9.42 × 10^−6^	3.71	miR-486-5p	5.46 × 10^−6^	−31.25
miR-126	1.78 × 10^−6^	−51.73	mir-572	1.41 × 10^−5^	2.35
-	-	-	miR-885-3p	7.96 × 10^−5^	6.77
-	-	-	miR-126	1.89 × 10^−5^	−29.22

FDR method was used.

**Table 8:** DE miRNAs found (using the non-FDR method) by comparing CD4+ with CD8+ T-cells for each disease group.

Disease State	miRNA	*p*-Value	Fold Change	Disease State	miRNA	*p*-Value	Fold Change
**Aviremic**	miR-151-3p	7.60 × 10^−3^	3.7	**Viremic**	miR-3185	5.68 × 10^−3^	−3.76
	miR-125b	3.93 × 10^−3^	4.97		miR-1909	1.90 × 10^−3^	−4.17
	miR-99a	1.39 × 10^−3^	7		miR-125a-5p	5.26 × 10^−3^	3.34
	miR-196a	7.99 × 10^−4^	−5.52		miR-2277	7.56 × 10^−4^	−2.63
	miR-152	6.15 × 10^−3^	−5.79		let-7e	1.69 × 10^−3^	3.05
**LTNP**	miR-151-5p	1.96 × 10^−5^	10.27		miR-125a-3p	4.31 × 10^−3^	3.45
	miR-1469	5.31 × 10^−3^	−2.73		miR-572	4.01 × 10^−3^	−3.27
	miR-486-5p	1.45 × 10^−4^	12.22		miR-99b	3.82 × 10^−3^	4.02
	miR-151-3p	7.78 × 10^−3^	4.3		miR-3195	1.30 × 10^−3^	−6.46
	miR-125b	9.64 × 10^−5^	13.35		miR-885-5p	2.92 × 10^−3^	−2.99
	miR-663	4.66 × 10^−3^	−3.43		miR-4286	8.22 × 10^−3^	−3.25
	miR-1909	5.81 × 10^−4^	−7.25	**Negative**	miR-1469	4.61 × 10^−3^	−3.25
	miR-1228 *	7.88 × 10^−3^	−3.34		miR-663	5.08 × 10^−3^	−4.09
	miR-885-3p	9.17 × 10^−4^	−4.15		miR-99a	9.83 × 10^−3^	7.11
	miR-1908	8.85 × 10^−3^	−3.12		miR-199a-5p	4.15 × 10^−3^	3.23
	miR-125a-5p	5.16 × 10^−5^	10.29		miR-125a-3p	3.35 × 10^−3^	6.1
	let-7e	1.19 × 10^−3^	4.12		miR-570	7.42 × 10^−3^	−2.8
	miR-125a-3p	9.93 × 10^−4^	6.03		mir-520f	3.35 × 10^−3^	2.43
	miR-92b *	1.76 × 10^−3^	−4.44		miR-921	5.02 × 10^−4^	2.05
	miR-1231	2.26 × 10^−3^	−5.56		miR-142-5p	1.40 × 10^−3^	3.39
	miR-99b	7.07 × 10^−4^	7.8		miR-1911 *	1.52 × 10^−3^	−2.25
	miR-146b-5p	5.95 × 10^−3^	4.64		miR-1256	7.49 × 10^−3^	−2.08
	miR-509-3p	1.65 × 10^−3^	5.31		mir-941-3_s	1.12 × 10^−3^	2.96
	miR-3195	4.66 × 10^−3^	−7.14		miR-127-3p	4.70 × 10^−3^	3.11
	miR-181a-2 *	6.21 × 10^−3^	−7.8		miR-628-5p	3.58 × 10^−3^	−3.9
	miR-3187	2.22 × 10^−3^	−2.98		mir-620	4.41 × 10^−3^	2.24
	miR-675	7.50 × 10^−3^	−2.63		miR-601	2.40 × 10^−3^	3.96
	miR-637	1.47 × 10^−3^	−2.5		miR-513a-5p	4.45 × 10^−3^	2.07
	miR-486-3p	8.41 × 10^−3^	4.41				
	miR-532-3p	3.21 × 10^−3^	3.64				
	miR-320e	6.89 × 10^−3^	2.67				

* Less functionally defined miRNAs. Non-FDR method. For each cell type, viremic *n* = 6, aviremic *n* = 5, LTNP *n* = 4 and negative *n* = 3. Negative fold change implicates downregulation in CD4+ T-cells (Upregulation in CD8+) and positive fold change implies upregulation in CD4+ T-cells (downregulation in CD8+).

**Table 9:** qRT-PCR results for miRNAs of high significance chosen for validation along with associated microarray results.

miRNA	Analysis Where Significant	Adjusted *p*-Value	Fold Change from Microarray	Hypothesis/Importance	Additional Significances from Non-FDR Analysis	Fold Change from qRT-PCR
126	CD4 LTNP *vs.* negative	1.76 × 10^−6^	−51.73	LTNP infection marker based on both CD4+ and CD8+	CD4 HIV+ *vs.* HIV− (CD4 viremic and LTNP *vs.* negative, LTNP *vs.* aviremic)	−103.76
	CD8 LTNP *vs.* negative	5.46 × 10^−6^	−31.25			−14.26
199a-5p	CD4 HIV+ *vs.* HIV−	1.22 × 10^−6^	−5.6	HIV Infection marker for CD4+ T-cells	CD4 HIV+ *vs.* HIV− (CD4 viremic, aviremic and LTNP *vs.* negative)	−209.48
	CD4 viremic *vs.* negative	4.61 × 10^−6^	−6.31			−2.73
	CD4 LTNP *vs.* negative	7.66 × 10^−6^	−6.85			−50.34

## Abbreviations

AIDS	acquired immunodeficiency syndrome
LTNP	long-term non-progressors
qRT-PCR	quantitative reverse transcriptase PCR
PCR	polymerse chain reaction
PBMC	peripheral blood mononuclear cells
miRNA	micro-ribonucleic acid
FDR	false discovery rate
PCA	principal component analysis
DE	differential expression
RIN	RNA integration number
BDL	below detectable levels

## Appendix

Supplementary results are presented below, for comparison with FDR method derived results presented in results section. These results are for assessing differential expression of miRNAs between cell types and different HIV disease groups.

**Table A1:** Differentially expressed miRNAs in HIV+ CD4+ and CD8+ cells compared to negative controls. Non-FDR method. Infected *n* = 15, Uninfected *n* = 3 for each cell type.

Cell type	miRNA	*p*-Value	Fold Change
CD4	miR-199a-5p	1.22 × 10^−6^	−5.60
	miR-720	6.61 × 10^−5^	3.69
	miR-1469	7.90 × 10^−3^	2.91
	miR-127-3p	9.48 × 10^−5^	−3.34
	miR-130a	2.10 × 10^−3^	−3.77
	miR-487b	5.00 × 10^−4^	−2.64
	miR-129-3p	3.00 × 10^−4^	−2.40
	miR-483-5p	7.00 × 10^−4^	−3.28
	miR-520f	3.00 × 10^−4^	−2.58
	miR-601	2.40 × 10^−3^	−2.77
	miR-126	5.40 × 10^−3^	−10.61
	miR-584	7.80 × 10^−3^	−2.78
	miR-432	5.80 × 10^−3^	−2.73
	miR-142-5p	1.20 × 10^−3^	−2.89
	miR-1280	2.00 × 10^−3^	2.33
	miR-139-5p	3.00 × 10^−3^	−2.78
	miR-3137	4.40 × 10^−3^	−2.01
	miR-606	3.50 × 10^−3^	−2.57
	miR-548i-4	3.50 × 10^−3^	−2.40
	miR-195	3.40 × 10^−3^	−4.21
CD8	let-7e	4.00 × 10^−3^	−3.02
	miR-941-3_s	5.83 × 10^−5^	2.77
	miR-1323	9.14 × 10^−5^	−3.72
	miR-4321	6.00 × 10^−3^	2.42
	miR-572	9.20 × 10^−3^	3.78
	miR-570	9.00 × 10^−4^	−3.17
	miR-1256	1.10 × 10^−3^	−2.12
	miR-4284	1.90 × 10^−3^	4.95
	miR-890	2.90 × 10^−3^	−2.58
	miR-589 *	9.10 × 10^−3^	2.57
	miR-603	6.70 × 10^−3^	−2.36

* Functionally undefined miRNA.

**Table A2:** Differentially expressed miRNAs of CD4+ T-cells in each disease state when compared to negative controls.

Disease State	miRNA	*p*-Value	Fold Change	Disease State	miRNA	*p*-Value	Fold Change
**LTNP**	miR-126	1.78 × 10^−6^	−51.73	**LTNP (cont’d)**	miR-1972	3.33 × 10^−3^	5.2
	miR-1469	3.85 × 10^−4^	4.24		miR-363	2.38 × 10^−3^	−11.54
	miR-151-3p	1.09 × 10^−3^	−7.4		miR-520f	7.11 × 10^−4^	−2.68
	miR-3141	1.28 × 10^−3^	2.06		miR-142-5p	2.40 × 10^−4^	−3.88
	miR-663	1.35 × 10^−3^	4.68		miR-130a	9.21 × 10^−3^	−3.61
	miR-99a	6.41 × 10^−3^	−7.04		miR-1207-5p	5.93 × 10^−3^	2.25
	miR-3185	4.20 × 10^−3^	6.19		miR-720	2.56 × 10^−4^	4.47
	miR-762	1.40 × 10^−3^	4.54		miR-302d	1.10 × 10^−4^	−2.19
	miR-1228 *	2.05 × 10^−3^	4.69		miR-199a-3p	4.81 × 10^−3^	−5.31
	miR-3196	2.80 × 10^−3^	3.62		miR-182	1.39 × 10^−3^	−2.69
	miR-149 *	9.18 × 10^−3^	5.74		miR-197	2.79 × 10^−3^	2.17
	miR-4281	4.99 × 10^−4^	4.12		miR-155 *	2.09 × 10^−3^	2.7
	miR-638	1.51× 10^−3^	3.42		miR-663b	4.23 × 10^−4^	4.61
	miR-1908	5.29 × 10^−3^	3.75		mir-151_x	1.97 × 10^−3^	−2
	miR-199a-5p	7.66 × 10^−6^	−6.85		miR-1260b	3.25 × 10^−3^	2.73
	miR-1915	1.24 × 10^−3^	4.16		miR-127-3p	6.51 × 10^−4^	−3.77
	miR-1268	3.76 × 10^−3^	2.94		miR-620	2.38 × 10^−3^	−2.25
	miR-2861	9.38 × 10^−4^	4.82		miR-548i-4	9.59 × 10^−3^	−2.41
	miR-1280	9.42 × 10^−6^	3.71		miR-100	3.02 × 10^−3^	−6.28
	miR-199b-3p	3.89 × 10^−3^	−4.6		miR-663b_x	3.31 × 10^−3^	2
	miR-195	6.18 × 10^−4^	−5.44		miR-487b	2.88 × 10^−3^	−2.84
	miR-1910	4.18 × 10^−3^	3.57		miR-584	3.31 × 10^−3^	−4.14
	miR-3175	6.93 × 10^−4^	8.47		miR-519e	4.35 × 10^−3^	−2.18
	miR-3163	7.54 × 10^−3^	2.75		miR-134	9.11 × 10^−3^	−2.15
**Viremic**	miR-126	4.42 × 10^−4^	−11.24	**Viremic Cont'd**	miR-3137	8.47 × 10^−4^	−2.56
	miR-1469	9.01 × 10^−4^	3.43		miR-487b	6.03 × 10^−3^	−2.41
	miR-663	3.15 × 10^−3^	3.66		miR-574-5p	9.77 × 10^−3^	2.64
	miR-99a	6.66 × 10^−4^	−10.45		miR-1308	7.95 × 10^−3^	2.71
	miR-1228 *	4.92 × 10^−3^	3.61		miR-601	1.98 × 10^−3^	−3.38
	miR-149 *	5.54 × 10^−3^	3.72		miR-23b *	8.30 × 10^−3^	−2.03
	miR-199a-5p	4.61 × 10^−6^	−6.31		miR-765	2.19 × 10^−3^	−2.35
	miR-1280	4.41 × 10^−4^	2.45		miR-1225-3p	5.74 × 10^−3^	2.11
	miR-195	8.72 × 10^−5^	−6.44		miR-214	9.80 × 10^−3^	−2.08
	miR-3175	1.40 × 10^−3^	6.29	**Aviremic**	miR-199a-5p	2.34 × 10^−4^	−4.13
	miR-483-5p	7.50 × 10^−5^	−4.28		miR-483-5p	1.20 × 10^−3^	−3.21
	miR-548q	1.04 × 10^−3^	−2.82		mir-520f	4.79 × 10^−3^	−2.13
	miR-520f	1.02 × 10^−4^	−2.96		miR-720	2.26 × 10^−3^	3.16
	miR-142-5p	1.49 × 10^−4^	−3.7		miR-127-3p	1.76 × 10^−3^	−3.14
	miR-130a	2.84 × 10^−4^	−5.83		miR-548i-4	8.26 × 10^−3^	−2.36
	miR-378	2.36 × 10^−3^	2.28		miR-129-3p	1.70 × 10^−3^	−2.47
	miR-720	4.92 × 10^−4^	3.69		miR-487b	2.33 × 10^−3^	−2.78
	miR-574-3p	2.43 × 10^−3^	3.19		miR-606	3.81 × 10^−3^	−3.05
	miR-23a *	8.67 × 10^−3^	3.48		miR-517a_x	7.91 × 10^−3^	−2.25
	miR-510	9.44 × 10^−3^	−2.59		miR-134	9.60 × 10^−3^	−2.06
	miR-127-3p	1.02 × 10^−3^	−3.24				
	miR-3172	9.57 × 10^−3^	−2.9				
	miR-548i-4	4.96 × 10^−3^	−2.44				
	miR-129-3p	7.83 × 10^−4^	−2.58				

* Non-FDR method. Viremic *n* = 6, aviremic *n* = 5, LTNP *n* = 4, negative *n* = 3.

**Table A3:** Differentially expressed miRNAs when disease classes of CD4 cells are intercompared.

CD4	miRNA	*p*-Value	Fold Change	Disease State	miRNA	*p*-Value	Fold Change	Disease State	miRNA	*p*-Value	Fold Change
Viremic *vs* Aviremic	miR-99a	1.68 × 10^−4^	−9.76	Viremic *vs* LTNP	miR-143	8.46 × 10^−3^	6.73	LTNP *vs* Aviremic	miR-223	3.66 × 10^−3^	−5.08
	miR-1307	3.11 × 10^−3^	2.15		miR-3163	1.10 × 10^−4^	−3.8		miR-197	7.76 × 10^−4^	2.19
	miR-744	8.69 × 10^−5^	2.48		miR-768-3p	9.30 × 10^−6^	−2.06		miR-1268	2.69 × 10^−3^	2.68
	miR-142-5p	5.24 × 10^−3^	−2.17		miR-1979	8.61 × 10^−3^	−3.39		miR-199b-3p	3.86 × 10^−4^	−5.56
	miR-574-3p	2.46 × 10^−3^	2.69		miR-1301	8.42 × 10^−3^	2.1		miR-142-5p	7.12 × 10^−3^	−2.27
	miR-25 *	1.73 × 10^−3^	2.05		miR-4284	7.78 × 10^−3^	−3.51		miR-199a-3p	3.15 × 10^−4^	−4.7
	miR-29c *	1.08 × 10^−4^	−2.39		miR-1973	3.77 × 10^−4^	−4.94		miR-363	7.80 × 10^−4^	−11.28
	miR-3175	2.37 × 10^−3^	4.41		miR-1201_s	8.31 × 10^−5^	−2.14		miR-155 *	6.37 × 10^−4^	2.69
	miR-195	2.82 × 10^−3^	−3.13		miR-548e	4.75 × 10^−3^	−2.07		miR-345	2.02 × 10^−3^	−5.92
	miR-29b	7.44 × 10^−3^	−5.34		miR-514b-5p	3.95 × 10^−4^	−3.45		miR-1280	2.29 × 10^−4^	2.46
	miR-30e *	1.47 × 10^−3^	−3.33		miR-663b	1.96 × 10^−3^	−3.02		miR-126	2.32 × 10^−5^	−18.55
	miR-19a	2.30 × 10^−3^	−2.11		miR-18a	8.66 × 10^−3^	4.77		miR-145	2.96 × 10^−4^	−25.89
	miR-23a *	7.59 × 10^−4^	4.18	LTNP *vs* Aviremic	miR-1469	9.73 × 10^−3^	2.39		miR-1972	1.81 × 10^−4^	6.99
Viremic *vs* LTNP	miR-2277	6.17 × 10^−3^	−2.34		miR-3185	4.43 × 10^−3^	4.91		miR-26a	1.72 × 10^−3^	−2.08
	miR-509-3p	7.77 × 10^−5^	−7.57		miR-99a	3.11 × 10^−3^	−6.57		miR-19b	1.18 × 10^−3^	-6.2
	miR-223	2.36 × 10^−3^	5.2		miR-151-3p	1.50 × 10^−3^	−5.46		miR-1975	1.94 × 10^−4^	2.31
	miR-510	7.00 × 10^−4^	−3.28		miR-762	4.66 × 10^−3^	3.17		miR-143	5.40 × 10^−4^	−15.36
	miR-155 *	7.69 × 10^−4^	−2.55		miR-3196	3.50 × 10^−3^	3		miR-3163	2.74 × 10^−3^	2.76
	miR-345	3.79 × 10^−5^	11.66		miR-1228 *	7.26 × 10^−3^	3.18		miR-3175	1.15 × 10^−3^	5.95
	miR-500	3.74 × 10^−3^	3.25		miR-4281	9.55 × 10^−3^	2.41		miR-29b	7.37 × 10^−3^	−6.41
	miR-145	1.16 × 10^−3^	15.52		miR-638	5.02 × 10^−3^	2.55		miR-30e *	4.53 × 10^−3^	−3.21
	miR-503	8.85 × 10^−3^	2.58		miR-1915	7.36 × 10^−3^	2.74		miR-30e	2.02 × 10^−3^	−8.1
	miR-424 *	4.85 × 10^−3^	4.44		miR-2861	8.03 × 10^−3^	2.9		miR-514b-5p	1.71 × 10^−3^	3.04
	miR-3152	2.93 × 10^−3^	−3.18		miR-149 *	8.41 × 10^−3^	3.24		miR-663b	2.30 × 10^−3^	3.09
	miR-1972	5.31 × 10^−5^	−7.9		miR-1910	1.72 × 10^−3^	3.47		miR-18a	4.15 × 10^−3^	−6.03
	miR-1975	2.03 × 10^−5^	−2.62		miR-509-3p	2.35 × 10^−4^	6.81		let-7f	9.09 × 10^−3^	−2.36

***** Non-FDR method. Viremic *n* = 6, Aviremic *n* = 5, LTNP *n* = 4, Negative *n* = 3.

**Table A4:** Differentially expressed miRNAs of CD8+ T-cells in each disease state when compared to negative controls.

CD8	miRNA	*p*-Value	Fold Change	Disease State	miRNA	*p*-Value	Fold Change	Disease State	miRNA	*p*-Value	Fold Change	Disease State	miRNA	*p*-Value	Fold Change
Viremic	miR-126	5.37 × 10^−3^	−6.26	Aviremic	miR-345	4.24 × 10^−3^	5.88	LTNP	miR-151-5p	7.06 × 10^−6^	−14.86	LTNP (cont’d)	miR-92b *	4.74 × 10^−4^	6.31
	miR-345	7.94 × 10^−4^	7.94		miR-181b	5.59 × 10^−4^	−2.54		miR-126	1.89 × 10^−5^	−29.22		miR-1979	3.18 × 10^−4^	8.16
	miR-541	8.79 × 10^−6^	−2.18		miR-223	9.75 × 10^−3^	4.7		miR-1469	1.42 × 10^−3^	3.55		miR-1231	3.43 × 10^−3^	5.82
	let-7e	5.30 × 10^−3^	−3.28		miR-3121	7.04 × 10^−3^	−2.37		miR-486-5p	5.46 × 10^−6^	−31.25		miR-572	2.25 × 10^−4^	8.3
	miR-3163	3.55 × 10^−3^	−2.82		miR-30e *	9.76 × 10^−3^	3.13		miR-1975	1.89 × 10^−3^	2.15		miR-99b	8.93 × 10^−3^	−5.15
	miR-548u	1.37 × 10^−3^	−2.12		miR-143	7.01 × 10^−3^	9.16		miR-151-3p	2.12 × 10^−4^	−10.35		miR-146b-5p	2.23 × 10^−3^	−6.51
	miR-572	4.74 × 10^−3^	4.14		miR-570	4.30 × 10^−4^	−3.58		miR-125b	5.61 × 10^−4^	−10.99		miR-4321	6.84 × 10^−4^	3.72
	miR-514b-5p	3.25 × 10^−3^	−2.96		miR-30e	5.68 × 10^−3^	7.42		miR-663	3.81 × 10^−3^	3.93		mir-1224	2.28 × 10^−3^	2.16
	miR-3121	7.81 × 10^−4^	−2.96		miR-29b	4.10 × 10^−3^	8.92		miR-3185	7.77 × 10^−3^	5.35		miR-1307	9.31 × 10^−3^	2.3
	miR-570	1.27 × 10^−3^	−3.95		mir-1298	2.42 × 10^−5^	−2.02		miR-762	2.57 × 10^−3^	4.11		miR-31	2.36 × 10^−3^	−9.14
	miR-3124	7.18 × 10^−3^	−4		miR-21	2.14 × 10^−3^	13.59		miR-1909	6.48 × 10^−3^	5.07		miR-4284	5.10 × 10^−4^	7.66
	mir-16-2	4.34 × 10^−4^	−2.19		miR-4284	5.78 × 10^−3^	4.4		miR-1228 *	1.44 × 10^−3^	4.99		miR-3188	4.37 × 10^−3^	4.7
	miR-3152	1.05 × 10^−3^	−4.14		miR-29c	7.63 × 10^−3^	10.28		miR-3196	4.44 × 10^−3^	3.37		miR-3187	8.03 × 10^−3^	2.72
	miR-4284	6.63 × 10^−3^	4.09		miR-1323	3.04 × 10^−4^	−4.13		miR-885-3p	7.96 × 10^−5^	6.77		miR-23b	2.01 × 10^−3^	−2.36
	miR-23a *	9.03 × 10^−3^	3.45		miR-4317	9.33 × 10^−3^	2.16		miR-149 *	3.55 × 10^−4^	6.8		miR-1225-5p	6.70 × 10^−4^	12.96
	miR-1323	1.72 × 10^−4^	−4.23		miR-26b	5.84 × 10^−3^	6.98		miR-4281	3.19 × 10^−3^	3.19		miR-3180-1_s	4.34 × 10^−3^	2.33
	miR-1256	2.62 × 10^−4^	−2.51		miR-548i-4_x	1.74 × 10^−3^	−2.1		miR-572	1.41 × 10^−5^	2.35		miR-885-5p	6.62 × 10^−3^	3.69
	miR-885-5p	7.07 × 10^−3^	3.31		miR-941-3_s	1.36 × 10^−3^	2.58		miR-638	6.71 × 10^−3^	2.79		miR-885	6.75 × 10^−3^	2.32
	miR-941-3_s	4.57 × 10^−4^	2.79		miR-27a *	5.75 × 10^−3^	6.21		miR-1908	5.91 × 10^−3^	3.68		miR-941-3_s	5.07 × 10^−4^	2.99
	miR-27a *	3.14 × 10^−3^	6.76		miR-27a	7.48 × 10^−3^	4.23		miR-1915	7.12 × 10^−3^	3.16		miR-637	4.18 × 10^−3^	2.4
	miR-603	4.66 × 10^−4^	−3.31		miR-488	8.19 × 10^−3^	−2.01		miR-1268	2.48 × 10^−4^	4.19		miR-939	2.55 × 10^−3^	3.91
	miR-488	1.99 × 10^−3^	−2.25		miR-140-5p	6.69 × 10^−3^	8.78		miR-2861	3.15 × 10^−3^	3.95		miR-30d	4.75 × 10^−3^	−3.91
	miR-628-5p	1.24 × 10^−3^	−3.79		miR-1263	5.08 × 10^−3^	−4.86		miR-125a-5p	1.87 × 10^−3^	−6.12		miR-346	4.63 × 10^−4^	6.9
	miR-16-2 *	8.92 × 10^−3^	−2.88		miR-98	7.70 × 10^−3^	2.46		miR-2277	2.02 × 10^−3^	3.17		miR-4322	2.12 × 10^−3^	3.1
	miR-1181	6.11 × 10^−3^	2.12		miR-374b	3.28 × 10^−3^	2.56		miR-17	2.03 × 10^−3^	−2.14		miR-3172	7.73 × 10^−3^	3.28
	miR-890	1.75 × 10^−3^	−3.03		miR-890	3.56 × 10^−3^	−2.87		miR-106a	2.71 × 10^−3^	−2.07		miR-665	7.41 × 10^−4^	6.02
	miR-200b *	6.99 × 10^−3^	−2.18		miR-331-5p	7.47 × 10^−3^	4.31		let-7e	1.49 × 10^−3^	−4.46		miR-92a-1 *	1.71 × 10^−3^	−4.54
	miR-335	4.81 × 10^−3^	−7.7						miR-20b	4.23 × 10^−4^	−8.91		miR-3162	3.53 × 10^−3^	4.33
	miR-1272	7.98 × 10^−3^	−3.12						miR-1910	1.35 × 10^−3^	4.28		miR-1300	2.24 × 10^−3^	6.4
									miR-3175	5.60 × 10^−3^	5.38		miR-1244	8.05 × 10^−3^	3.26

* Non-FDR method. Viremic *n* = 6, aviremic *n* = 5, LTNP *n* = 4, negative *n* = 3.

**Table A5:** Differentially expressed miRNAs when disease classes of CD8+ cells are intercompared.

CD8	miRNA	*p*-Value	Fold Change	Disease State	miRNA	*p*-Value	Fold Change
Viremic *vs* Aviremic	miR-181b	3.63 × 10^−5^	2.64	Viremic *vs* LTNP (cont’d)	miR-3126-3p	1.29 × 10^−4^	−2.18
	miR-1915	8.81 × 10^−3^	2.42		miR-501-3p	2.70 × 10^−3^	3.77
	miR-548q	6.20 × 10^−3^	−2.05		miR-502-3p	1.23 × 10^−3^	3.25
	miR-603	9.70 × 10^−3^	−2.05		miR-345	3.45 × 10^−4^	7.75
Viremic *vs* LTNP	miR-151-5p	2.22 × 10^−5^	8.22		miR-1289-2	7.24 × 10^−5^	−2.31
	miR-151-3p	1.11 × 10^−3^	5.41		miR-202 *	7.47 × 10^−3^	−2.53
	miR-762	9.96 × 10^−3^	−2.71		miR-126	9.69 × 10^−3^	4.67
	miR-1228 *	7.96 × 10^−3^	−3		miR-145	1.62 × 10^−3^	14.08
	miR-92b *	3.74 × 10^−3^	−3.47		miR-378c	7.77 × 10^−3^	2.49
	miR-486-5p	4.53 × 10^−6^	19.01		miR-494	2.21 × 10^−3^	−3.39
	miR-532-3p	7.63 × 10^−3^	2.86		miR-132	1.90 × 10^−3^	3.3
	miR-885-3p	2.17 × 10^−5^	−5.95		miR-635	2.08 × 10^−3^	−2.98
	miR-3124	2.42 × 10^−3^	−4.29		miR-30d	4.95 × 10^−3^	3.15
	miR-637	1.88 × 10^−3^	−2.26		miR-378	3.94 × 10^−3^	2.03
	miR-3141	4.88 × 10^−5^	−2.26		miR-92a-1 *	7.78 × 10^−4^	4.01
	miR-146b-5p	2.71 × 10^−3^	4.7		miR-450b-5p	7.85 × 10^−3^	−2.36
	miR-149 *	7.94 × 10^−3^	−3.13		miR-130b	4.37 × 10^−3^	2.03
	miR-532-5p	7.31 × 10^−4^	3.85		miR-1975	6.75 × 10^−4^	−2.05
	miR-652	1.09 × 10^−3^	2.19		miR-629	9.89 × 10^−3^	6.42
	miR-500 *	1.47 × 10^−3^	2.34		miR-665	4.03 × 10^−3^	−3.51
	miR-20b	1.60 × 10^−4^	7.5		mir-548q	1.55 × 10^−3^	−3.4
	miR-16-2 *	9.86 × 10^−3^	−2.59		miR-3163	8.73 × 10^−3^	−2.31
	miR-223	7.10 × 10^−3^	4.19		miR-106b	2.95 × 10^−3^	4.13
	miR-106a	4.98 × 10^−5^	2.44		miR-509-3-5p	1.22 × 10^−3^	−2.37
	miR-17	4.70 × 10^−5^	2.48		miR-1979	1.77 × 10^−3^	−4.46
	miR-20a	5.96 × 10^−4^	3.48		miR-548x	5.20 × 10^−3^	−3.13
	miR-501-5p	3.44 × 10^−3^	2.35		miR-3172	5.90 × 10^−3^	−2.83
	miR-3121	9.34 × 10^−4^	−2.64		miR-28-5p	8.09 × 10^−3^	3.22
	miR-1226 *	2.02 × 10^−3^	−3.54		miR-27a *	3.74 × 10^−3^	5.51
	miR-1268	7.37 × 10^−4^	−2.98		miR-1973	3.88 × 10^−3^	−3.47
	miR-4253	5.05 × 10^−3^	−2.1		miR-3128_x	6.06 × 10^−3^	−2.45
	miR-548u	1.79 × 10^−4^	−2.3				
LTNP *vs* Aviremic	miR-1909	1.40 × 10^−3^	5.57	LTNP *vs* Aviremic	miR-17	7.47 × 10^−5^	−2.49
	miR-151-5p	2.08 × 10^−5^	−9.03		miR-20a	4.10 × 10^−5^	−5.1
	miR-125b	1.99 × 10^−3^	−6.33		miR-501-5p	8.41 × 10^−3^	−2.2
	miR-1469	6.34 × 10^−4^	3.35		let-7d *	6.75 × 10^−4^	−2.23
	miR-663	8.00 × 10^−4^	4.18		miR-4322	9.04 × 10^−3^	2.28
	miR-3185	7.88 × 10^−4^	6.92		miR-664 *	8.44 × 10^−3^	−2.38
	miR-1908	7.06 × 10^−4^	4.31		miR-21	2.60 × 10^−3^	−10.41
	miR-151-3p	6.66 × 10^−4^	−6.34		miR-1268	4.17 × 10^−5^	4.28
	miR-762	9.57 × 10^−5^	5.5		miR-939	5.12 × 10^−3^	3
	miR-3196	2.49 × 10^−4^	4.25		miR-548u	1.23 × 10^−3^	2.05
	miR-1228 *	9.00 × 10^−5^	6.2		miR-199b-3p	4.34 × 10^−4^	−5.46
	miR-4281	1.11 × 10^−4^	4.14		miR-199a-3p	5.41 × 10^−3^	−4.24
	miR-92b *	1.20 × 10^−3^	4.37		miR-146a	6.01× 10^−3^	−3.17
	miR-638	4.71 × 10^−5^	4.38		miR-363	3.14 × 10^−3^	−7.99
	miR-1915	1.25 × 10^−4^	4.71		miR-1225-5p	6.03 × 10^−3^	5.74
	miR-486-5p	1.90 × 10^−5^	−16.05		miR-3126-3p	2.70 × 10^−4^	2.14
	miR-885-3p	5.09 × 10^−5^	5.7		miR-501-3p	2.58 × 10^−3^	−4
	miR-2861	8.69 × 10^−5^	5.53		miR-502-3p	6.25 × 10^−4^	−3.71
	miR-637	4.30 × 10^−3^	2.15		mir-326	8.02 × 10^−4^	2.09
	miR-3141	6.97 × 10^−6^	2.65		miR-345	2.36 × 10^−3^	−5.74
	miR-146b-5p	4.17 × 10^−4^	−7.07		miR-320e	8.38 × 10^−3^	−2.47
	miR-149 *	4.05 × 10^−5^	7.49		miR-548q	3.22 × 10^−3^	−2.38
	mir-3185	8.85 × 10^−3^	2.23		mir-1289-2	4.14 × 10^−5^	2.48
	miR-532-5p	1.05 × 10^−3^	−3.86		miR-1207-5p	9.97 × 10^−4^	2.41
	miR-3188	2.72 × 10^−3^	4.23		miR-126	9.90 × 10^−4^	−8.34
	miR-572	1.72 × 10^−3^	4.58		miR-500	4.10 × 10^−3^	−3.35
	miR-1910	1.50 × 10^−3^	3.53		mir-885	2.94 × 10^−3^	2.28
	miR-500 *	3.87 × 10^−3^	−2.2		miR-145	1.30 × 10^−3^	−16.72
	miR-20b	1.34 × 10^−4^	−8.38		miR-660	8.64 × 10^−3^	−2.33
	miR-223	5.85 × 10^−3^	−4.62		miR-494	5.62 × 10^−4^	4.33
	miR-106a	1.16 × 10^−4^	−2.38		miR-132	5.84 × 10^−3^	−2.95
	miR-30d	4.93 × 10^−4^	−4.66		miR-30e *	1.51 × 10^−3^	−3.78
	miR-27a	5.93 × 10^−4^	−5.92		miR-1979	1.32 × 10^−4^	7.32
	miR-140-3p	2.24 × 10^−3^	−2.06		miR-30c	4.30 × 10^−4^	−2.56
	miR-339-3p	2.06 × 10^−3^	−3.79		miR-3172	8.10 × 10^−3^	2.82
	miR-31	2.45 × 10^−4^	−11.48		miR-346	3.84 × 10^−3^	3.85
	miR-26a	7.81 × 10^−4^	−2.21		miR-26b	3.91 × 10^−3^	−6.56
	miR-1263	9.55 × 10^−3^	3.77		miR-30e	9.59 × 10^−4^	−9.65
	miR-30b	5.32 × 10^−4^	−12.55		miR-128	7.31 × 10^−3^	−5.32
	miR-29c	7.64 × 10^−3^	−8.5		miR-194	1.50 × 10^−3^	−7.99
	miR-19b	3.54 × 10^−4^	−7.8		miR-19a	2.13 × 10^−3^	−2.31
	miR-17 *	8.67 × 10^−3^	−5.42		miR-28-5p	8.44 × 10^−4^	−4.92
	miR-4270	6.97 × 10^−4^	2.68		miR-210	7.28 × 10^−3^	−4.66
	miR-1975	6.73 × 10^−6^	2.95		miR-27a *	7.32 × 10^−3^	−5.07
	miR-143	7.91 × 10^−3^	−7.39		miR-23b	3.02 × 10^−4^	−2.5
	miR-769-5p	8.46 × 10^−4^	−6.87		miR-25	6.61 × 10^−4^	−2.31
	miR-28-3p	1.72 × 10^−3^	−2.25		miR-625	4.34 × 10^−3^	−2.26
	miR-629	1.15 × 10^−3^	−12.53		miR-4317	2.44 × 10^−3^	−2.33
	miR-29a	4.11 × 10^−3^	−4.22		miR-923	2.67 × 10^−3^	3.15
	miR-106b	3.94 × 10^−4^	−6.18		let-7f	2.37 × 10^−3^	−2.79
	miR-22	7.87 × 10^−3^	−4.23		let-7g	2.73 × 10^−4^	−2.22
	miR-195	6.29 × 10^−3^	−3.12		miR-182	1.62 × 10^−3^	−2.35
	miR-29b	3.99 × 10^−3^	−7.52				

* Non-FDR method. Viremic *n* = 6, Aviremic *n* = 5, LTNP *n* = 4, Negative *n* = 3.

**Table A6:** qRT-PCR raw amplification values.

Sample	miR-199a	miR-126	U6B	Sample	miR-199a	miR-126	U6B	Sample	miR-199a	miR-126	U6B
V1 CD4 (1)	31.45	27.85	18.16	AV1 CD4 (1)	29.68	26.13	17.12	LTNP2 CD4 (1)	36.16	32.71	17.19
V1 CD4 (2)	30.69	28.52	17.33	AV1 CD4 (2)	29.42	26.07	17.01	LTNP2 CD4 (2)	33.88	31.94	15.51
V1 CD8 (1)	32.34	29.16	16.45	AV1 CD8 (1)	30.38	27	16.62	LTNP2 CD8 (1)	33.19	29.53	15.7
V1 CD8 (2)	31.5	28.84	15.59	AV1 CD8 (2)	29.75	26.99	16.04	LTNP2 CD8 (2)	33.26	29.41	15.66
V2 CD4 (1)	31.36	27	16.79	AV2 CD4 (1)	30.66	27.18	16.56	LTNP3 CD4 (1)	35.12	30.62	16.34
V2 CD4 (2)	30.78	28.65	17.92	AV2 CD4 (2)	30.45	27.53	16.27	LTNP3 CD4 (2)	34.48	30.96	16.59
V2 CD8 (1)	33.16	29.12	15.93	AV2 CD8 (1)	31.59	28.65	16.37	LTNP3 CD8 (1)	35.09	33.4	17.11
V2 CD8 (2)	32.2	29.62	16.17	AV2 CD8 (2)	30.74	27.62	16.06	LTNP3 CD8 (2)	34.67	32.64	16.6
V3 CD4 (1)	30.72	25.73	16.55	AV3 CD4 (1)	29.87	No Ct	16.15	LTNP4 CD4 (1)	35.17	33.15	15.36
V3 CD4 (2)	30.34	25.68	16.75	AV3 CD4 (2)	30.3	26.24	15.74	LTNP4 CD4 (2)	34.89	32.93	15.85
V3 CD8 (1)	33.17	28	15.98	AV3 CD8 (1)	33.04	28.85	16.43	LTNP4 CD8 (1)	31.87	28.82	15.12
V3 CD8 (2)	33.57	28.3	16.6	AV3 CD8 (2)	33.55	29.27	17.06	LTNP4 CD8 (2)	31.2	29.22	15.01
V4 CD4 (1)	31.65	26.99	17.63	AV4 CD4 (1)	31.22	27.37	16.95	Neg1 CD4 (1)	29.86	26.12	16.42
V4 CD4 (2)	30.4	26.65	16.68	AV4 CD4 (2)	30.29	26.92	16.62	Neg1 CD4 (2)	29.68	25.67	15.76
V4 CD8 (1)	30.83	27.16	16.15	AV4 CD8 (1)	31.37	27.73	14.53	Neg1 CD8 (1)	32.43	28.76	16.46
V4 CD8 (2)	30.12	26.68	15.59	AV4 CD8 (2)	30.6	27.6	15.39	Neg1 CD8 (2)	32.97	28.65	16.79
V5 CD4 (1)	33.92	29.44	17.48	AV5 CD4 (1)	32.53	27.87	15.42	Neg2 CD4 (1)	29.86	26.09	15.62
V5 CD4 (2)	33.41	29.08	17.23	AV5 CD4 (2)	31.65	27.69	15.8	Neg2 CD4 (2)	29.75	26.25	15.74
V5 CD8 (1)	33.4	28.12	16.26	AV5 CD8 (1)	31.62	27.29	16.11	Neg2 CD8 (1)	31.8	27.93	15.63
V5 CD8 (2)	32.48	27.83	15.43	AV5 CD8 (2)	30.83	26.91	16.81	Neg2 CD8 (2)	31.62	27.89	15.63
V6 CD4 (1)	33.34	29.47	16.91	LTNP1 CD4 (1)	35.31	33.16	16.5	Neg3 CD4 (1)	25.99	22.73	14.86
V6 CD4 (2)	32.81	28.92	16.43	LTNP1 CD4 (2)	34.5	32.92	15.86	Neg3 CD4 (2)	26.35	22.53	14.28
V6 CD8 (1)	33.08	28.69	16.64	LTNP1 CD8 (1)	32.84	29.91	13.92	Neg3 CD8 (1)	30.74	26.2	18.15
V6 CD8 (2)	33.48	29.66	17.12	LTNP1 CD8 (2)	32.3	30.01	14.88	Neg3 CD8 (2)	30.82	26.91	17.48

## References

[1] Fowler L., Saksena N.K. (2013). Micro-RNA: New players in HIV-pathogenesis, diagnosis, prognosis and antiviral therapy. AIDS Rev..

[2] Houzet L., Jeang K.T. (2011). MicroRNAs and human retroviruses. Biochim. Biophys. Acta.

[3] Calin G.A., Sevignani C., Dumitru C.D., Hyslop T., Noch E., Yendamuri S., Shimizu M., Rattan S., Bullrich F., Negrini M. (2004). Human microRNA genes are frequently located at fragile sites and genomic regions involved in cancers. Proc. Natl. Acad. Sci. USA.

[4] Croce C.M. (2009). Causes and consequences of microRNA dysregulation in cancer. Nat. Rev. Genet..

[5] Calin G.A., Dumitru C.D., Shimizu M., Bichi R., Zupo S., Noch E., Aldler H., Rattan S., Keating M., Rai K. (2002). Frequent deletions and downregulation of micro-RNA genes miR15 and miR16 at 13q14 in chronic lymphocytic leukemia. Proc. Natl. Acad. Sci. USA.

[6] Yeung M.L., Bennasser Y., Myers T.G., Jiang G., Benkirane M., Jeang K.T. (2005). Changes in microRNA expression profiles in HIV-1-transfected human cells. Retrovirology.

[7] Houzet L., Yeung M.L., De Lame V., Desai D., Smith S.M., Jeang K.T. (2008). MicroRNA profile changes in human immunodeficiency virus type 1 (HIV-1) seropositive individuals. Retrovirology.

[8] Huang J., Wang F., Argyris E., Chen K., Liang Z., Tian H., Huang W., Squires K., Verlinghieri G., Zhang H. (2007). Cellular microRNAs contribute to HIV-1 latency in resting primary CD4+ T lymphocytes. Nat. Med..

[9] Witwer K.W., Watson A.K., Blankson J.N., Clements J.E. (2012). Relationships of PBMC microRNA expression, plasma viral load, and CD4+ T-cell count in HIV-1-infected elite suppressors and viremic patients. Retrovirology.

[10] Zhu L., Qui C., Wang J., Yuan S., Zhang X., Xu J. Poster MOPE026—MicroRNA profile identifies different outcomes of disease progression at later stage of HIV infection. Proceedings of the XIX IAS Conference.

[11] Cohen M., Shaw G., McMichael A., Haynes B. (2011). Acute HIV-1 Infection. N. Engl. J. Med..

[12] Koup R.A., Safrit J.T., Cao Y., Andrews C.A., McLeod G., Borowsky W., Farthing C., Ho D.D. (1994). Temporal association of cellular immune responses with the initial control of viremia in primary human immunodeficiency virus type 1 syndrome. J. Virol..

[13] Borrow P., Lewicki H., Hahn B.H., Shaw G.M., Oldstone M.B.A. (1994). Virus-specific CD8+ cytotoxic T-lymphocyte activity associated with control of viremia in primary human immunodeficiency virus type 1 infection. J. Virol..

[14] Sanghvi V.R., Steel L.F. (2011). The cellular TAR RNA binding protein, TRBP, promotes HIV-1 replication primarily by inhibiting the activation of double-stranded RNA-dependent kinase PKR. J. Virol..

[15] Bignami F., Pilotti E., Bertoncelli L., Ronzi P., Guilli M., Marmirolli N., Magnani G., Pinti M., Lopalco L., Mussini C. (2012). Stable changes in CD4+ T lymphocyte miRNA expression after exposure to HIV-1. Blood.

[16] Noorbakhsh F., Ramachandran R., Barsby N., Ellestad K.K., LeBlanc A., Dickie P., Baker G., Hollenberg M.D., Cohen E.A., Power C. (2010). MicroRNA profiling reveals new aspects of HIV neurodegeneration: Caspase-6 regulates astrocyte survival. FASEB J..

[17] Bennasser Y., Jeang K.T. (2006). HIV-1 Tat interaction with Dicer: Requirement for RNA. Retrovirology.

[18] Rossi R.L., Rossetti G., Wenandy L., Curti S., Ripamonti A., Bonnal R.J., Birolo R.S., Moro M., Crosti M.S., Gruarin P. (2011). Distinct microRNA signatures in human lymphocyte subsets and enforcement of the naive state in CD4+ T-cells by the microRNA miR-125b. Nat. Immunol..

[19] Allantaz F., Cheng D.T., Bergauer T., Ravindran P., Rossier M.F., Ebeling M., Badi L., Reis B., Bitter H., D’Asaro M. (2012). Expression profiling of human immune cell subsets identifies miRNA-mRNA regulatory relationships correlated with cell type specific expression. PLoS ONE.

[20] Nathans R., Chu C.Y., Serquina A.K., Lu C.C., Cao H., Rana T.M. (2009). Cellular microRNA and P bodies modulate host-HIV-1 interactions. Mol. Cell.

[21] Sun G., Li H., Wu X., Covarrubias M., Scherer L., Meinking K., Luk B., Chomchan P., Alluin J., Gombart A.F. (2012). Interplay between HIV-1 infection and host microRNAs. Nucleic Acids Res..

[22] Tan Gana N.H., Onuki T., Victoriano A.F., Okamoto T. (2012). MicroRNAs in HIV-1 infection: An integration of viral and cellular interaction at the genomic level. Front. Microbiol..

[23] Chiang K., Sung T.L., Rice A.P. (2012). Regulation of cyclin T1 and HIV-1 Replication by microRNAs in resting CD4+ T lymphocytes. J. Virol..

[24] Triboulet R., Mari B., Lin Y.L., Chable-Bessia C., Bennasser Y., Lebrigand K., Cardinaud B., Maurin T., Barbry P., Baillat V. (2007). Suppression of microRNA-silencing pathway by HIV-1 during virus replication. Science.

[25] Jopling C., Yi M., Lancaster A., Lemon S., Sarnow P. (2005). Modulation of hepatitis C virus RNA abundance by a liver-specific microRNA. Science.

